# Supplementary material for: Diversification of mandarin citrus by hybrid speciation and apomixis
Source: Nat Commun. 2021 Jul 26;12:4377. doi: 10.1038/s41467-021-24653-0 (PMC8313541; doi:10.1038/s41467-021-24653-0)
Supplement: Supplementary file 1 — Supplementary Information [file 41467_2021_24653_MOESM1_ESM.pdf]

Diversification of mandarin citrus by hybrid speciation and apomixis

Wu *et al*

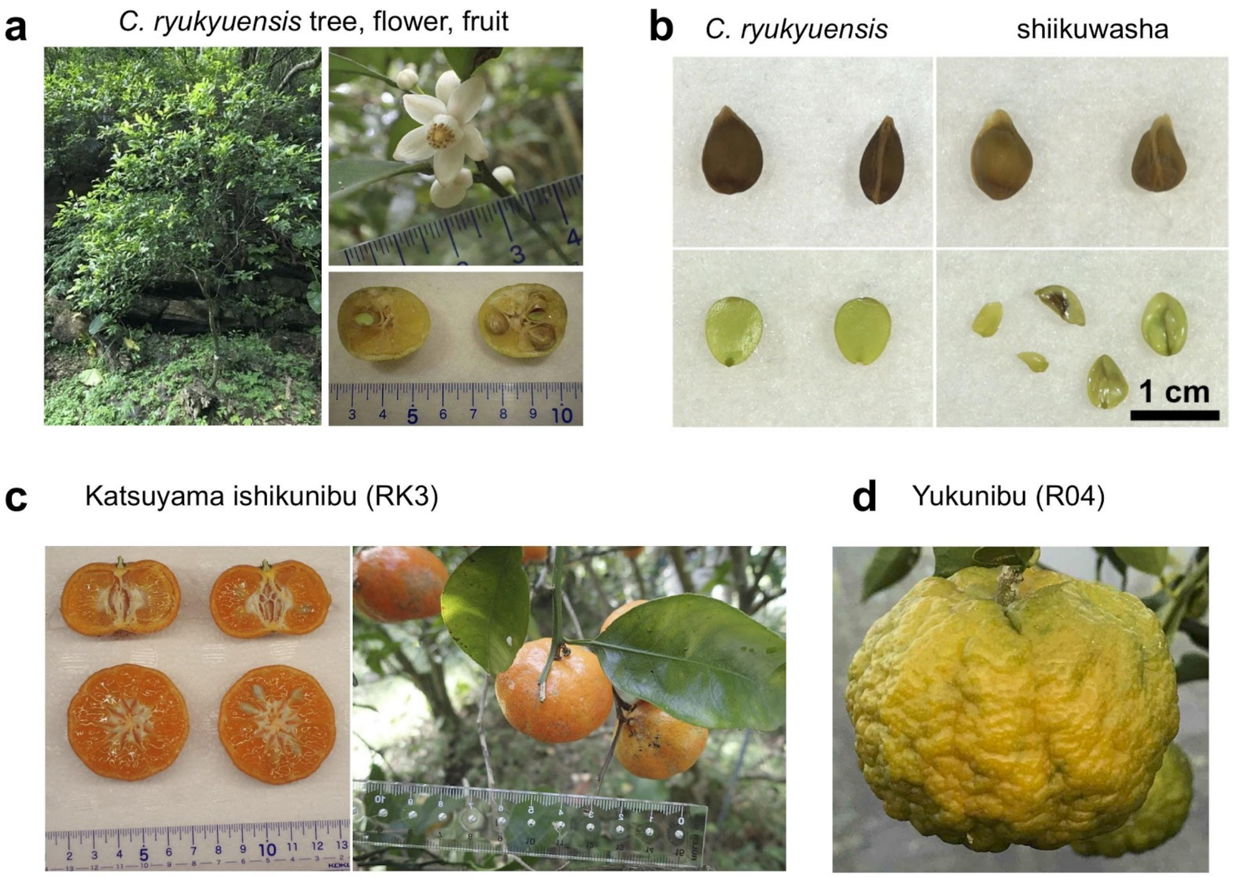

Supplementary Figure 1. Okinawa citrus collection. a) *C. ryukyuensis* tree, flower and cut fruit. **b)** Seeds of *C. ryukyuensis* (monoembryonic, left panels) and shiikuwasha (*C. depressa*, polyembryonic, right panels). Top panels: seeds with seed coat; bottom panels: seed coat removed, revealing embryos. **c)** Fruits and leaf of the Katsuyama ishikunibu (RK3), the mainland Asian mandarin parent of shiikuwasha. **d**) fruit of the Yukunibu accession R04.

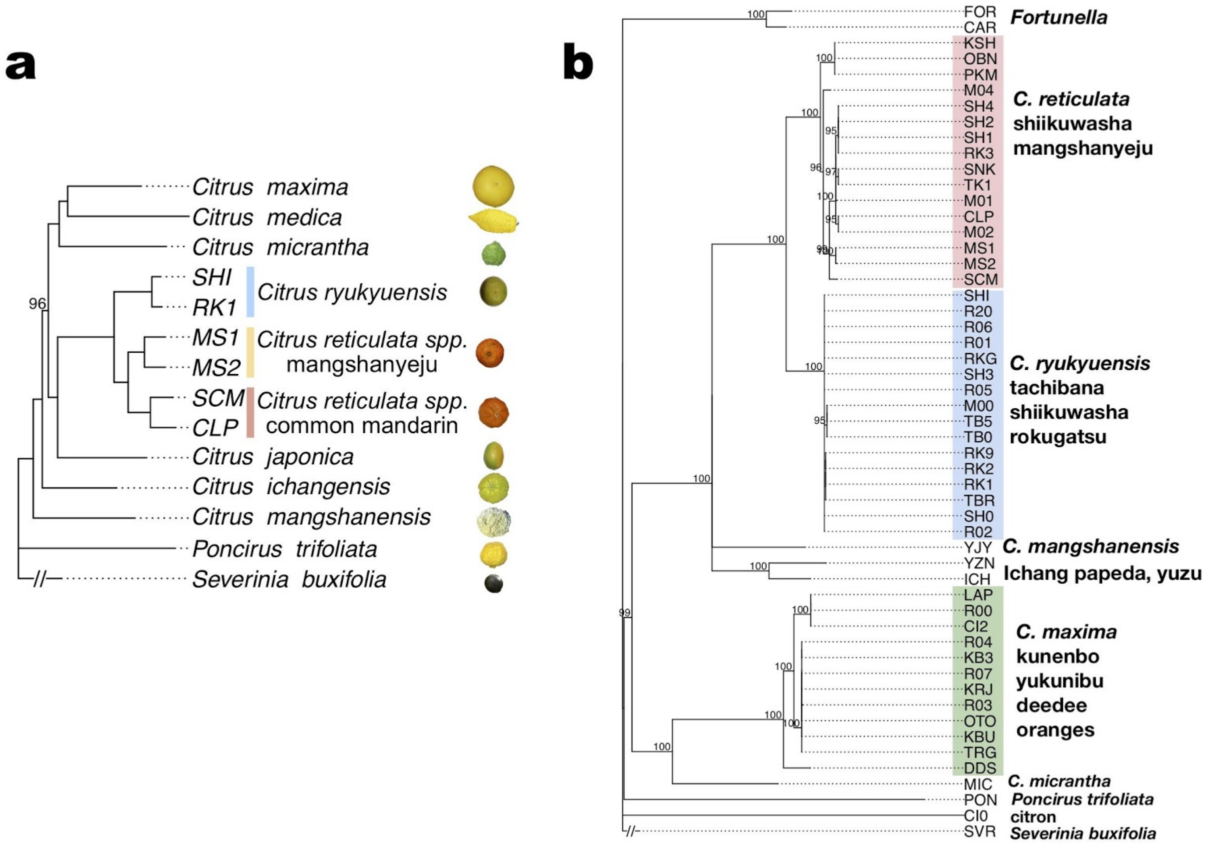

Supplementary Figure 2. Nuclear and chloroplast genome phylogeny of east Asian citrus. **a)** Nuclear genome maximum likelihood phylogenetic tree of progenitor Asian citrus species, rooted with *Severinia buxifolia* (aka Chinese box orange). Bootstrap support values other than 100% are shown. *Citrus japonica* is also known as *Fortunella* or kumquat. Note that *Citrus mangshanensis* is a distinct species from mangshanyeju (*C. reticulata*). **b)** Chloroplast genome maximum likelihood tree is reconstructed using RAxML[1]. Bootstrap support values more than 90% are shown. The tree is rooted with Chinese box orange (*Severinia buxifolia*).

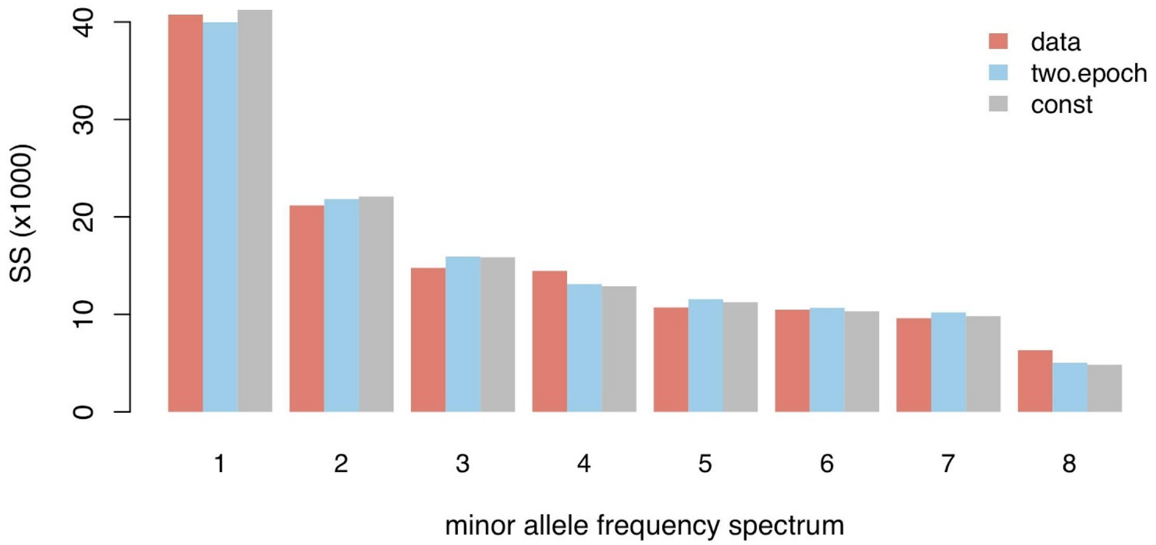

Supplementary Figure 3. **Allele frequency spectrum (AFS) of n=8 accessions of *C. ryukyuensis* from Okinawa based on single nucleotide polymorphisms.** The folded AFS is computed by excluding genomic regions with inter-specific admixture and using segregating sites (SS) with no missing data among the 8 accessions. For comparison, the expected AFS for a panmictic (random mating) population with constant effective population size (gray color) and of a two-epoch model (light blue) are also shown. Based on the likelihood ratio test, the constant effective population size model is rejected (*P*-value=1.1×10^{-20}) in favor of a two-epoch model with a population bottleneck (**Supplementary Note 3**). Source data are provided as a Source Data file.

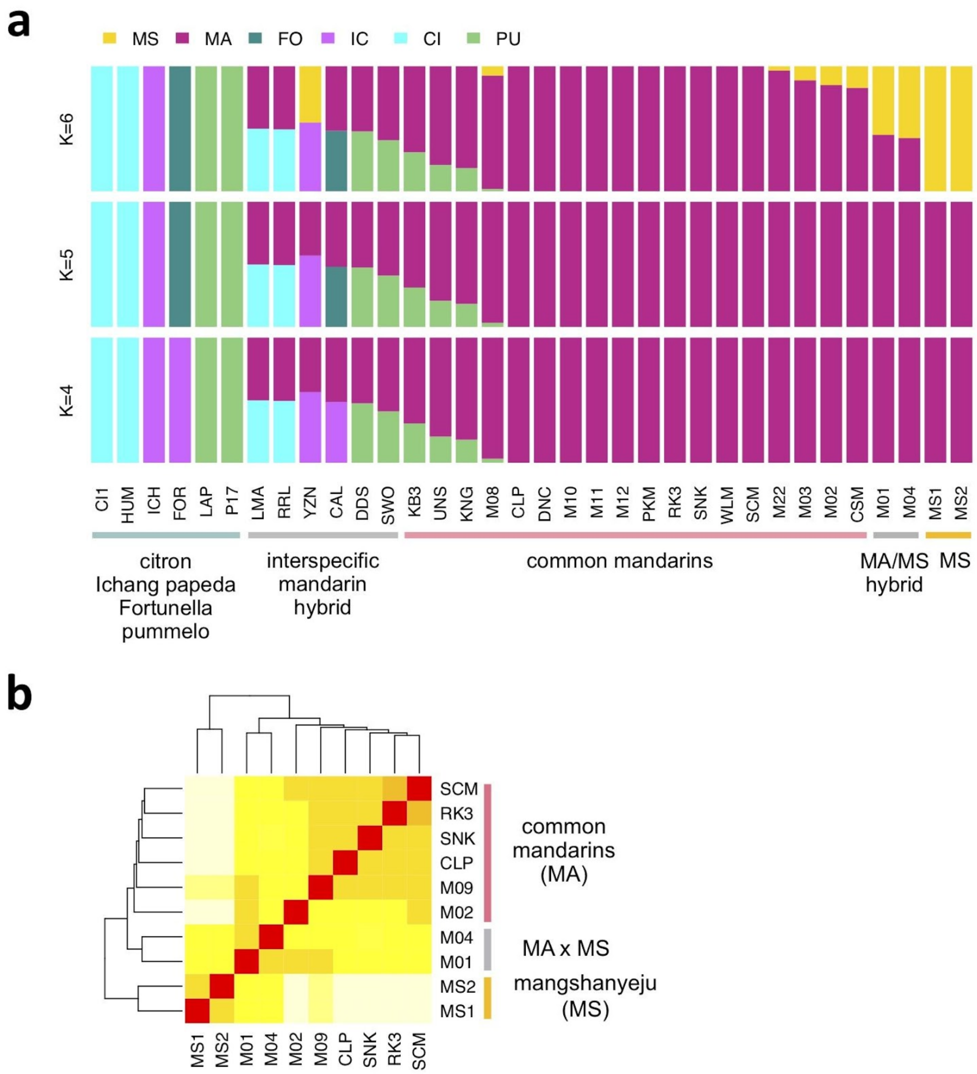

Supplementary Figure 4. Mangshanyeju (MS) represents a distinct mandarin population from common mandarins (MA). a) ADMIXTURE[2] analysis of 34 diverse mainland Asian citrus varieties derived from five progenitor species. This figure complements **Fig. 1b** by including additional citrus species and interspecific mandarin hybrids but excluding citrus with *C. ryukyuensis* ancestry. Ancestry proportions are shown for K=4-6 genetic clusters, with K=6 having the lowest cross validation error. Two citrus species (ICH=Ichang papeda, FOR=*Fortunella*) are not resolved at K=4. At K=5, all five species are resolved. At K=6, differentiation between mangshanyeju and common mandarin subspecies is observed. PU=pummelo, CI=citron, IC=Ichang papeda, FO=*Fortunella*, MA=common mandarin and MS=mangshanyeju. Two wild mandarins (M01=Daoxian wild mandarin and clonal relatives, M04=Suanpangan) are hybrid between MS and MA. Yuzu (YZN) (*C. junos*) is shown to be the only known interspecific hybrid with MS as paternal parent. Note that ADMIXTURE fails to detect the small amount of pummelo admixture in type 2 mandarins[3] except M08 (bendizao). Similarly, ADMIXTURE is not sensitive to small amount of MS admixture in some common mandarins as identified by local ancestry inference (**Fig. 1b**). **b)** Heat map of pairwise genomic distance (D) shows mangshanyeju (MS) as a distinct population of *C. reticulata,* separate from common mandarins (MA). Source data underlying Supplementary Figure 4a are provided as a Source Data file.

a

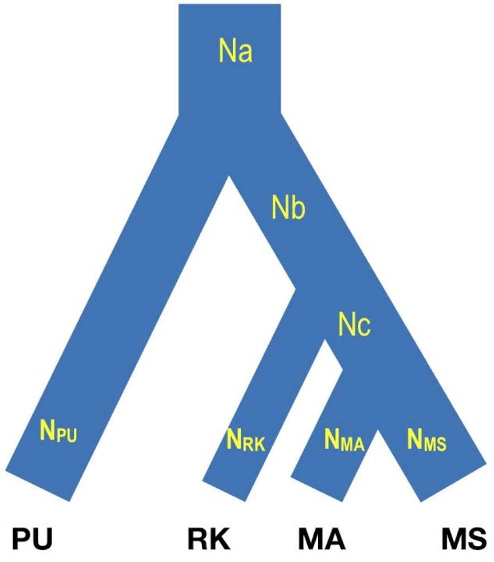

b

Parameters	Model estimate
PU split time (Mya)	6-7.5
RK split time (Mya)	2.2-2.8
MS/MA split (Mya)	1.4-1.7
Na (x1000)	482-602
Nb (x1000)	156-195
Nc (x1000)	166-207
N_{MA} (x1000)	84-105
N_{MS} (x1000)	104-130
N_{RK} (x1000)	56-70
N_{PU} (x1000)	148-185

Supplementary Figure 5. East Asian citrus speciation. a) A minimal 4-population model with 10 parameters for the divergence of pummelos (PU), *C. ryukyuensis* (RK), common mandarins (MA) and mangshanyeju (MS). **b)** Inferred divergence times and effective population sizes based on modeling 4-dimensional allele frequency spectrum (AFS) using moments[4]. Na, Nb, *etc.* are corresponding effective population sizes. The PU/MA divergence time is set to 6-7.5 Mya based on a previous estimate[3] and citrus leaf fossil record. A generation time of 10 years is used for effective population size estimate. Genomic regions with inter-population admixture are excluded in the computation of joint AFS.

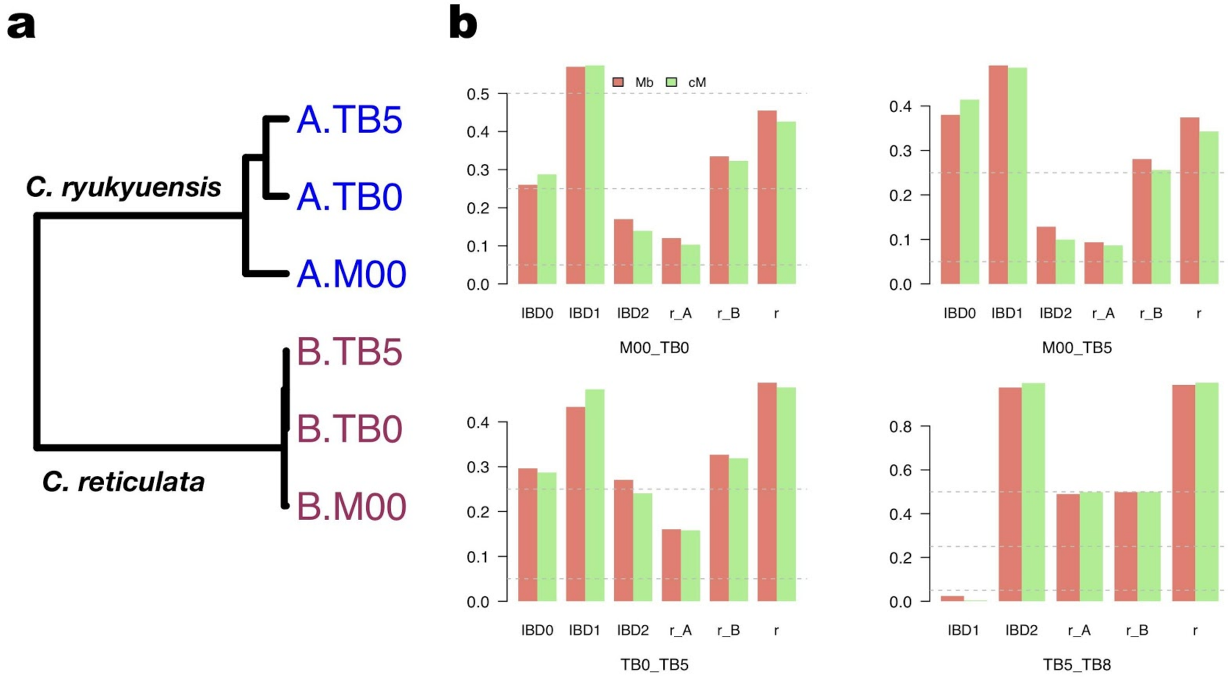

Supplementary Figure 6. **Tachibana haplotype tree and relatedness. a**) Haplotype tree of three tachibana genotypes at the apomixis locus (200kb flanking *CitRKD1*, Chr1: 25.4-25.6 Mb). The prefixes 'A' and 'B' denote *C. ryukyuensis* and *C. reticulata* ancestry respectively. The three distinct *C. ryukyuensis* haplotypes indicate that multiple *C. ryukyuensis* individuals were at the origin of tachibana. By contrast, genome wide haplotype sharing pattern suggests that the *C. reticulata* ancestry of tachibana could be attributed to a single mainland Asian mandarin through a complex admixture process. **b)** Pairwise relatedness analysis based on genome wide haplotype sharing using regions where at least one tachibana is *C. ryukyuensis/C. reticulata* hybrid. The coefficient of relatedness between different genotypes (r=r_A + r_B) receives significantly more contribution from the *C. reticulata* haplotype (r_B) than from the *C. ryukyuensis* haplotype (r_A). Also shown as a control is the clonal pair TB5 and TB8 with r=1. Genomic proportions of sharing no, one, or two haplotypes in each pairwise comparison are denoted by IBD0, IBD1, IBD2 respectively. Salmon and light green bars denote estimates based on sequence size and genetic map distance respectively.

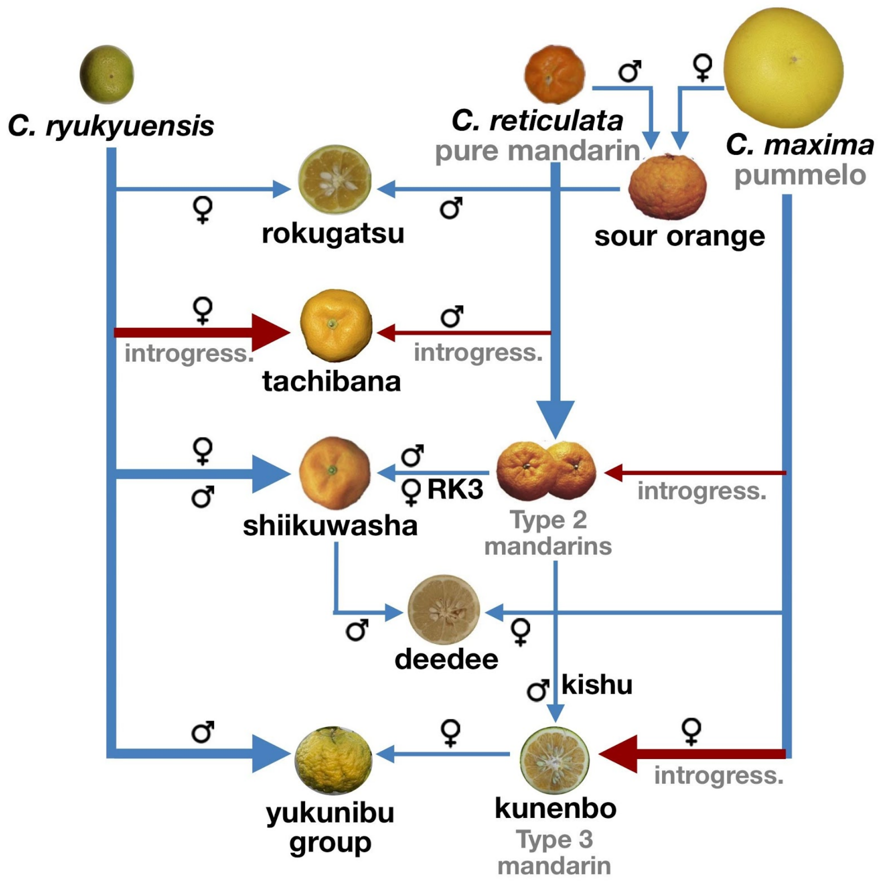

Supplementary Figure 7. Genealogy of major native Ryukyuan and mainland Japanese citrus types derived from three ancestral species. Thick lines denote ancestry involving multiple individuals from a population, whereas a thin line stands for single individual ancestry. Simple crosses and complex introgressions are shown in blue and red respectively. Type 2 mandarins are characterized by small amount of pummelo admixture whereas type 3 have more pummelo introgression[3]. The shiikuwashas are half-sibs sharing the same mainland Asian mandarin parent (RK3) but different *C. ryukyuensis* parents. Kunenbo is the seed parent of the yukunibu group. The Ryukyu sour orange (deedee) is a hybrid between pummelo and shiikuwasha.

a

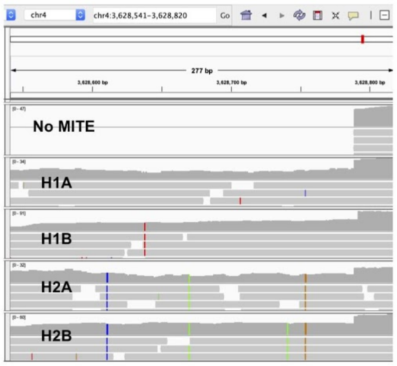

b

MITE haplotypes	H1A (SWO)	H1B (SSO)	H2A (MS1)	H2B (YZN)
Chr4:3628611	T	T	C	C
Chr4:3628638	C	T	C	C
Chr4:3628670	G	G	A	A
Chr4:3628741	G	G	G	A
Chr4:3628754	A	A	G	G

c d

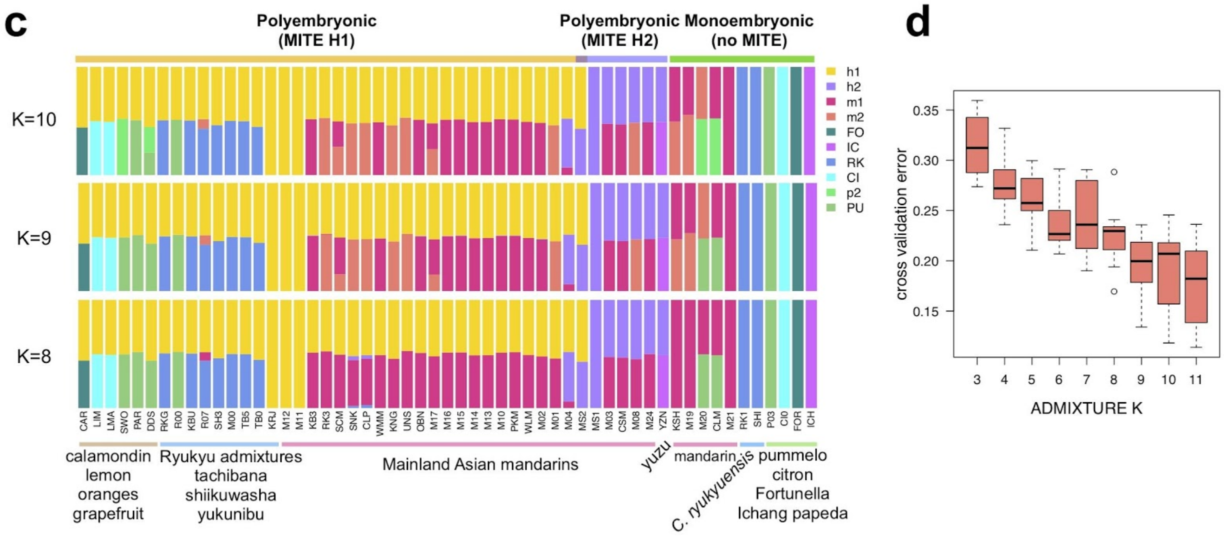

Supplementary Figure 8. Diversity and ancestry of citrus apomixis alleles. a) MITE transposon haplotypes in the promoter of *CitRKD1* gene regulating citrus apomixis. Integrative genomics viewer[5] display of five allele types based on sweet orange reference sequence[6]. The five representative citrus accessions containing the five allele types are *C. ryukyuensis* (RK1, no MITE), kunenbo mandarin (KB1, H1A), daidai sour orange (DDS, H1B), mangshanyeju (MS1, H2A), and yuzu (YZN, H2B). **b)** Five segregating single nucleotide polymorphisms among the MITE haplotypes. The MITE haplotypes form two haplogroups H1 (containing H1A, H1B types) and H2 (containing H2A, H2B types) separated by three fixed differences at positions Chr4: 3628611, Chr4:3628670 and Chr4:3628754. (see **Fig. 4a** for an illustration). Also listed are representative genomes containing each haplotype (SWO=sweet orange, SSO=sour orange, MS1=mangshanyeju, YZN=yuzu). **c**) Mangshanyeju ancestry at the polyembryonic locus (200kb region flanking *CitRKD1* gene). This figure complements **Fig. 4b** by including additional ADMIXTURE plots for K=8-10. All three plots (K=8-10) show consistently that nucellar embryony is associated with the mangshanyeju wild mandarin ancestry, with further population differentiation (m1, m2) in common mandarins at K=9. At K=10, population differentiation (p2, PU) in the pummelo is also observed. Other population labels follow **Fig. 4b**. For each K, 20 runs were performed and the run with the lowest cross-validation error was used. **d)** Boxplot of ADMIXTURE cross validation errors based on 20 runs for each K. Median, first and third quartiles are denoted by the thick line and bar limits respectively, with whisker denoting 1.5x the

interquartile distance for each K. Source data underlying Supplementary Figure 8c are provided as a Source Data file.

Supplementary Note 1. Historical and cultural aspects of citrus in Japan and Ryukyu Islands

Native citrus types in southwestern Japan

Two citrus types are believed to have been present in southwestern Japan before recorded history[1]:

- *C. tachibana* (Makino) T. Tanaka, commonly known as tachibana, grows wild on the Pacific side of the southwest of Japan's main islands, with populations also described in the Ryukyu Islands[7].
- *C. depressa* (Hayata) is indigenous to the Ryukyu islands, where it is known as shiikuwasha in Okinawan dialect; other common names include 'flat lemon' due to the shape of fruit and its very sour juice.

Both tachibana and shiikuwasha exhibit nucellar embryony, which often produces seeds with multiple somatically-derived embryos (polyembryony)[8-10]. Somatic embryos are clonally derived from maternal tissues, and often outcompete the zygotic (sexual) embryo. Thus, nucellar embryony typically results in apomictic propagation by seed, which presumably is responsible for the widespread distribution of clones of tachibana and shiikuwasha even in distant locations. Nevertheless, there are multiple genotypes of tachibana and shiikuwasha, a paradox that we resolve here with our hybrid origin scenario for these types.

While tachibana and shiikuwasha are widely recognized as distinct citrus types, their taxonomic status depends on the classification system used. There are two widely used classical systematizations of citrus taxonomy. The renowned Japanese citrologist Tyōzaburō Tanaka described 162 distinct citrus species, and based on his extensive analysis of citrus diversity classified tachibana and shiikuwasha as distinct species[11]. Tanaka also described[12] tanibuta in Okinawa, which he regarded as a variety of tachibana, *C. tachibana* (Makino) Tanaka var. *attenuata.* In contrast, the American botanist Walter Swingle favored a scheme with fewer fundamental species. Swingle[13] noted that tachibana is of 'great antiquity in Japan [which] seems to preclude it being a hybrid or 'chance seedling' of recent origin'. Remarking on its similarity to mandarin citrus, he concluded that 'It probably should be considered as a satellite species of *C. reticulata* and somewhat closely related to it'. Swingle further suggested that 'The Shekwasha has just the characters that would result from hybridizing of the native *C. tachibana* of Taiwan and the Ryukyu Islands with some form of the mandarin orange (*C. reticulata*) from the Chinese mainland'.

In addition to these classical studies based on morphological characters, the pioneering work of Yamamoto and colleagues[7,14,15] has brought molecular data to bear on the nature of tachibana and shiikuwasha and their possible relationships. In their analysis of citrus chloroplast genomes, Yamamoto *et al.* found that tachibanas, including Ryukyu tachibana (tanibuta), carry 'type 4' citrus cpDNA. In contrast, shiikuwashas can carry 'type 4' or 'type 5' citrus cpDNA, suggesting the possibility that shiikuwashas formed two or more times[7]. *C. tachibana* from the main islands of Japan also show considerable diversity[16].Yamamoto further noted that while tanibuta has been considered to belong to *C. tachibana* based on morphology[17], it is more closely related to *C. depressa* based on analysis of nuclear sequence-related amplified polymorphism (SRAP)

markers. Finally, Yamamoto suggested a hybrid origin of shiikuwasha based on high heterozygosity in the Got-2 (glutamate oxalacetate transaminase) isozyme[14,18]. Regarding the origin of tachibana, Shimizu and collaborators have suggested, based on genome-wide genotyping data, that tachibanas are interspecific hybrids of mandarin and an unspecified other species that is possibly related to citron[19]. Our prior genome sequence-based analysis[3] of tachibana grouped it with *C. reticulata*, but this relationship is refined in the present work using the extensive set of Japanese and Ryukyuan citrus genomes reported here.

In the remainder of **Supplementary Note 1** we provide background information on the history and cultural significance of tachibana and shiikuwasha. See **Supplementary Notes 6, 7 and 8** for background on other citrus and relationship to our findings.

Tachibana mandarins

Tachibana mandarins are prized for their fragrance and as ornamentals, and they are often planted outside of Shinto holy sites. The earliest written record of tachibana is found in the ***Man'yōshū***, a collection of classical Japanese poetry compiled circa 750 AD[20], and the tree appears in the earliest mythology of Japan[20]. In the poetry number 125 in ***Man'yōshū*** composed by Mikata no Sami, a portrait of a man taking a meditative walk in shadows of Tachibana trees is presented[21] . English translations of several poems referencing tachibana blossoms and garlands can be found in Tanaka's 'Citrus Fruits of Japan'[20].

Another possible poetic mention of tachibana can be found in the ***Kojiki*** ('Record of Ancient Matters'), a compilation of myths, legends, and songs completed around 720 C.E.[20,22]. A legend written in *Kojiki* II, compiled by Oono Yasumaro, tells that Emperor Suinin (29-99 CE, according to legend) sent Tajimamori to Tokoyo no Kuni, a legendary paradise, to seek Tokijiku no Kagu no Konomi, 'fruit with eternal fresh smell'. This 'fruit with eternal fresh smell' is interpreted by Sullivan *et al.*[22] as 'wild orange' but may refer to tachibana. The same Chinese/Japanese character is used for both.

Wild tachibana forests have been reported in Wakayama, Tokushima, Kochi, Yamaguchi, Saga, Miyazaki, and Kagoshima prefectures in western and southern mainland of Japan[23]. Tachibana is believed to be indigenous to the southwestern regions of mainland Japan. According to Tanaka[20], 'the primeval forest of Tachibana is found at Tsuro-mura, in the country of Aki, prefecture of Kochi, [which] has been officially registered as a Natural Monument Reservation of Japan'. Tanaka also notes that tachibana is abundant in the province of Hyuga, the fabled location of Tachibana no Odo that is mentioned in Japanese creation myths.

As noted above, tachibana trees produce polyembryonic seeds by nucellar embryony, a process that generates embryos that are maternal clones. Tachibana can therefore be propagated asexually by seed, as well as by grafting.

Shiikuwasha

Shiikuwasha, also known as flat lemon or Hirami lemon, are economically and culturally important acidic fruit trees grown in the Ryukyu islands, where they are believed to be indigenous[24]. Shiikuwasha is valued for its acidic properties, and is traditionally used to bleach

textiles[25]. It is also consumed as fruit and juice, and it is thought to contain high levels of health-promoting compounds.[17] Like tachibana, shiikuwasha can be propagated asexually by seed containing nucellar embryos, as well as by grafting. It is sometimes used as a rootstock and in rootstock breeding[26].

The earliest written record of native Ryukyuan citrus is found in the ***Omoro Sōshi***, a 16th~17th century compilation of chants from the Ryukyu Kingdom. The exact period when these chants were composed is unknown. Considering that the collection of the chants began during the era of King Sho-Shin (1477 CE~1527 CE), the chants from rural areas such as those found in ***Omoro Sōshi*** Vol. 2 are presumed to be composed sometime between the 3rd and 15th centuries[27].

In these compilations, native citrus are referred to as 'kuganii' or 'kunibu' which are the common names used today for different varieties of shiikuwasha[25]. Shiikuwasha is an inclusive term used to merchandize this fruit. The earliest record of 'kuganii' and 'kunibu' in the ***Omoro Sōshi*** are:

- **Kuganii.** In chant number 75 (34) in the ***Omoro Sōshi*** Vol. 2, there is a description of a foremost priestess dancing under a beautiful 'kuganii' tree[28,29]. ***Omoro Sōshi*** Vol. 2 is one of the collections of chants composed in the rural areas in Okinawa.
- **Kunibu.** In chant number 984 (3) in the ***Omoro Sōshi*** Vol. 14, there is a description of 'kunibu' bead necklace[29]. Fuyū Iha reported that young Ryukyuan or Okinawan girls had worn a small kunenbo beads necklace as a talisman against evils until circa 1890[30]. ***Omoro Sōshi*** Vol. 14 is a collection of chants about anecdotes of local heroes and well-known happenings of that time.

The Okinawan dialect word 'kunibu' is usually translated into standard Japanese as 'kunenbo'.[28] This does not necessary mean, however, that 'kunibu' in ***Omoro Sōshi*** is used exclusively to refer to 'kunenbo'. To explore this distinction, we consulted with local citrus experts with extensive knowledge of citrus traditions in Okinawa, who confirmed that the word 'kunibu' is often used in Okinawan dialect as a collective term for citrus in general. Similar term usage is also found in Amami region in Kagoshima, Japan, where the term for kunenbo is 'tookunibu', meaning 'China kunibu'. This information is based on interviews with Mr. Hideyasu Kinjo (age 70) and Mr. Hideo Ikemiyagi (age 81) conducted on 24 September 2019 and 17 October 2019 in Motobu Town, Okinawa, Japan.

Tanibuta (Ryukyu tachibana)

As noted above, Tyōzaburō Tanaka[12] described a variety of Ryukyuan citrus locally called tanibuta ('tani' = seeds, 'buta' = many or big in Okinawan dialect) as 'Ryukyu tachibana'. The name tanibuta is not widespread among Okinawans. Tanaka interpreted this monoembryonic variety as a type of tachibana and designated it *C. tachibana* var *attenuata* based on the attenuated base of its leaves. He noted that the fruits are 'slightly sweet and completely acidless', without remarking that this is in distinct contrast to the sour fruit of tachibana. Other authors have included tanibuta in their characterizations of Japanese citrus. Yamamoto, for example, noted that tanibuta types have the same cpDNA as tachibana (type 4), a cpDNA that is also shared with some shiikuwasha[7]. The designation of tanibuta as a variety of tachibana has not been challenged, though molecular differences between tanibuta and tachibana have been noted[7].

T. Tanaka described 'tanibuta' in his Japanese book of 1957 and reported the same contents in English[12] as 'A revision of Ryukyu Rutaceae-Aurantoidea Revision Aurantiacearum X'. 'Leaves very thick, in large ones lamina measuring 7.2 × 3.7 cm. (petiole 0.9 cm. long) obovate, base distinctly attenuate into wingless petiole. Fruits small, depressed-globose, mostly less then 3cm. in diam., largest 3.9 cm. across, generally resuming rectangular shape, apex deeply concave, base naked, devoid of oil cells and not accompanying radial grooves or striations; calyx very much raised, more or less large, body part broad and concave toward the stem, rather regularly 5 lobed. In transverse section of the fruit, rind thin, detached from the pulp ball, segments all free, 6-8 in number, outer ends much rounded, acutish at base; pulp little in amount, very light colored, melting, slightly sweet and completely acidless; pulp vesicles few in number, very short and mostly sessile. Seeds large, plump, obovate, smooth, slightly compressed, adherent to the carpel wall, testa extremely thick, tegmen light colored, especially so at chalaza, containing green monoembryo'. We note that while our accessions called tanibuta generally match Tanaka's phenotypic description, our accessions are acidic, in contrast with Tanaka's statement that tanibuta is 'completely acidless'.

Below we find that a collection of monoembryonic types, including accessions described as tanibuta, comprise a distinct genetic cluster that is separate from both tachibana and shiikuwasha. We show that this cluster represents a previously unrecognized sexual species that we call *C. ryukyuensis* sp. nov. We find that tachibana and shiikuwasha are both hybrid taxa. Each hybrid individual in these taxa has one *C. ryukyuensis* parent. Shiikuwasha and tachibana have different non-*C. ryukyuensis* parents of Chinese origin.

Supplementary Note 2. Sampling and sequencing Ryukyuan and mainland Japanese citrus

Supplementary Data 1 and 2 show a complete list of all accessions we collected and sequenced as part of this project. For sequencing, we collected shoots or young leaves from each sample tree. **Supplementary Data 3** shows the source of previously published genome sequencing data used in our analysis. These collections are organized into groups below based in part on relationships inferred from genome data. Here we provide additional background information on these samples.

Tachibana samples and genotypes

We analyzed two previously sequenced tachibana:

- TBM was obtained from the University of California Riverside collection. This tree was donated to the US by T. Tanaka. TBM was previously sequenced by UC Riverside[3]. We found that five of the tachibana (TB0, TB3, TB4, TB6, TB7) collected in Japan that we sequenced in the present project are clonally related to TBM.
- M00 (accession 8L) is a tachibana sequenced by Wang *et al.*[31]. No information given about its source. M00 and TBM have distinct genotypes. We found no clones of M00 in our sampling of mainland Japanese tachibana.

We sampled 7 additional tachibana from mainland Japan. While five of these were clonally related to TBM, we also discovered a third distinct tachibana genotype.

- TB0 is a garden accession from Hashimoto, Shingu City, Wakayama Prefecture, south of Osaka, provided by Shuji Takino. This tree was cultivated from the seeds that came from the mountain close to Hanano Iwaya, a world heritage site in Mie Prefecture, east of Wakayama, but outside the protected area. We find that it is clonally related to TBM.
- TB3 and TB4 were wild trees sampled in Arima, Kumano City, Mie Prefecture, near the original parental wild tree of TB0 but outside the protected area. We found by sequencing that these two accessions are also clonal relatives of TB0 and TBM.
- TB6 is a garden accession from Shingu, Shingu City, Wakayama Prefecture, provided by Shuji Takino. The origin of this garden accession was unknown. Genome sequencing shows that it is also clonally related to TBM.
- TB7 is a wild tree sampled from Suno, Kumano City, Mie Prefecture, provided by Hirofumi Hamada. It is also a clone of TBM.
- TB5 and TB8 are garden accessions from Nikou, Nachikatsuura Town, Wakayama Prefecture, provided by Naoe Ooe. The wild origin of these trees is unknown. Genome sequencing shows that these two accessions are clonally related to each other, but distinct from TBM and M00. TB5 and TB8 therefore represent a third tachibana genotype.

Shiikuwasha and wild Ryukyu samples

To broadly sample shiikuwasha and wild relatives, we collected (1) named varieties of shiikuwasha, (2) unnamed local shiikuwasha trees from personal gardens and small farms, and (3) wild shiikuwasha-like trees from various sources as described below. As described in the main text and **Supplementary Note 3** we found that several wild shiikuwasha-like trees in fact represent a previously unrecognized sexually reproducing species similar to accessions referred

to as tanibuta ('big (or many) seeds' in Okinawan dialect; we note that this name is not universally used among citrus growers in Okinawa, who do not cultivate tanibuta types). We have called this taxon *C. ryukyuensis*. Many shiikuwasha accessions are clonally related (**Supplementary Data 1**).

Named cultivated varieties of shiikuwasha

There are many named cultivated varieties of shiikuwasha. These are clonally propagated using the polyembryonic seeds produced by nucellar embryony. Some varieties have specific morphological characters and have been given local names. The highest commercial value are kuganii shiikuwasha; 'kuganii' means 'gold' in Okinawan dialect. Varieties of kuganii-type shiikuwashas named according to region or source, e.g., Oogimi-kuganii, Katsuyama-kuganii and Izumi-kuganii. Shiikuwashas called Njakuganii ('Nja' means bitter), Kaabishii, Kaachii ('kaa' means fruit skin, 'bishii' means thin, 'achii' means thick), Akakunibu (red citrus), Ishikunibu, Yama Mikanguwa ('yama' means mountain, 'mikan' means citrus, 'guwa' means small), and Nakamoto seedless are also locally recognized with their specific characters. As noted above, 'ishikunibu' is a general descriptor in Okinawan dialect that translates roughly as 'hard or strange citrus'.

- Njakuganii shiikuwasha (SH0), Kaabishii shiikuwasha (SH1), Kaachii shiikuwasha (SH2) and Kuganii shiikuwasha (SH3) were obtained from Asahikawa, Nago City, northern area of mainland Okinawa, provided by Hirofumi Higa, a citrus grower found through a local market in Onna Village, Okinawa.
- Nakamoto seedless shiikuwasha (SH4) was provided by Okinawa Prefectural Agricultural Research Center (OPARC), Nago, Nago City, Okinawa, through Fumimasa Mitsube. By genome analysis we find that SH4 is related to SH2 as described below in **Supplementary Note 4**.
- Oogimi-kuganii shiikuwasha (SH5), Katsuyama-kuganii shiikuwasha (SH6), and two Akakunibu shiikuwasha (RK4 and RK5) were obtained from Katsuyama, Nago City, Okinawa, provided by Katsuyama Shiikuwasha Co., Ltd., through Yoshikatsu Yamakawa.
- Izumi-kuganii shiikuwasha (R15) was obtained from Izumi, Motobu Town, in the northwestern area of the Motobu peninsula in mainland Okinawa, provided by Eijyun Zamami.
- Yama Mikanguwa shiikuwasha (R02) was obtained from Katsuyama, Nago City, Okinawa, provided by Masakazu Nakazato.
- We also included in our analysis a shiikuwasha accession from the collection of the Florida Dept. of Agriculture and Consumer Services, Division of Plant Industry, Citrus Budwood Registration Bureau (SHF). This tree is a second-generation seedling descendant of a shiikuwasha tree brought to Florida by Walter Swingle in 1927, given to him by the Tanaka Citrus Experimental Station at Tanushimaru, Kyushu, Japan. Our genome analysis showed that it is clonally related to SH1 (kaabishii shiikuwasha).

Local unnamed shiikuwasha

In addition to named varieties, we also obtained samples from local growers and public areas in Okinawa Prefecture.

- SH7 was obtained from Isagawa, Nago City, Okinawa, provided by Hideyasu Kinjo.

- TB1 and TB2 were obtained from the farm of Hiroshi Kobashigawa in Oogimi, Oogimi Village, northern area of mainland Okinawa. TB1 and TB2 are clonally relatives of SH0. (They were initially accidentally labeled 'TB' and are not tachibana.)
- RK6 was obtained from Oku, Kunigami Village, at the northern tip of Okinawa Island, by Sachiko Miyagi. The tree RK6 is more than 200 years old
- RK7 and RK8 were provided by Hiroshi Kobashigawa from his citrus farm in Oogimi, Oogimi Village, Okinawa. The trees of RK7 and RK8 are more than 100 years old.
- R01 was obtained from Katsuyama, Nago City, Okinawa, provided by Katsuyama Community Center, through Hideki Yamashiro.
- R07 was obtained from Kaneshi, Nakijin Village, in the northwestern area of Motobu peninsula on mainland Okinawa, provided by Toru Yonamine.
- R08 and R09 were collected from wild trees in Mt. Shioyafuji of Oshikawa, Oogimi Village, Okinawa.
- R10 and R11 were collected from wild trees in Mt. Awadake of Katsuyama, Nago City, Okinawa.
- R12 was collected from a wild tree in Mt. Daisekirin, a theme park called 'Daisekirinzan', managed by Nanto Co., Ltd. in the Yambaru National Park in Ginama, Kunigami Village, Okinawa, through Katsurou Kinjyou (Permission to collect these samples was granted by the Ministry of the Environment of Japan; Kan Kyu Chi Oki Kyo Dai 1904251 Go [環九地沖許第1904251号].)
- R13 was obtained from Genga, Nago City, Okinawa, provided by Kaori Miyazato.
- R14 was obtained from Imadomari, Nakijin Village, Okinawa, provided by Yukihiro Shimabukuro.
- R16, R17 and R18 were obtained from Katsuyama, Nago City, Okinawa, provided by Hideaki Oshiro.

Unusual shiikuwasha and wild Ryukyu citrus

In addition to cultivated and wild shiikuwasha, we also collected and sequenced atypical shiikuwashas and wild relatives. Some of these produced monoembryonic seeds, that is, they reproduce sexually, in contrast to the generally polyembryonic seeds of tachibana and shiikuwasha that arise via nucellar embryony. These are tanibuta types' ('tani' = seed, 'buta' = many or big) that match Tanaka's general description[12], although (1) our tanibuta types are acidic, and (2) they lack the characteristics that led Tanaka to infer polyploidy We also collected several accessions from wild mountainous forests in the northern part of Okinawa. Our atypical shiikuwasha-like accessions included some described as 'ishikunibu', found on small farms. (In Okinawan dialect, 'ishi' = stone, 'kunibu' = citrus, indicating that it is an atypical citrus with a small hard fruit.) Ishikunibu is a colloquial term that is also sometimes applied to specific shiikuwasha varieties. By genome analysis we show that these accessions are members of a previously unrecognized sexual species that we call *C. ryukyuensis*.

- The accession RK3, which we call 'Katsuyama ishikunibu', was an atypical shiikuwasha provided by Katsuyama Shiikuwasha Co., Ltd., Katsuyama, Nago City, Okinawa, through Yoshikatsu Yamakawa. It is not a tanibuta type. This garden accession has some similar characters with the accession 'Ishikunibu' in the picture book of Tanaka[23] (1948). By genome analysis we find that this accession is not a shiikuwasha but a mainland Asian mandarin; this genotype is a parent of all shiikuwasha in our collection.

- SHI, RK1, RK2 and RK9 were 'ishikunibu' types obtained from Oogimi, Oogimi Village, Okinawa, provided by Hiroshi Kobashigawa. These trees were collected from his farm but are not shiikuwasha and are presumed to be derived from seedlings from wild citrus trees in the surrounding forest.
- TBR was obtained from Isagawa, Nago City, Okinawa, provided by Hideyasu Kinjo. This garden accession was cultivated from the grafting that came from Mt. Shioyafuji of Oshikawa, Oogimi village, Okinawa. It is the same tanibuta tree analyzed with several markers by Inafuku-Teramoto *et al.*[17]
- R20 was collected from the wild tree in Mt. Shioyafuji, Oshikawa, Oogimi village, Okinawa. The tree was different from the TBR tree that was collected.
- R05 and R06 were collected from wild trees in Mt. Daisekirin, a theme park called 'Daisekirinzan', managed by Nanto Co., Ltd. in the Yambaru National Park in Ginama, Kunigami Village, Okinawa, through Katsurou Kinjyou. We received permission to collect these samples from Ministry of the Environment of Japan; Kan Kyu Chi Oki Kyo Dai 1904251 Go [環九地沖許第1904251号]).

Notes on other citrus samples

We also collected other citrus samples in Okinawa, including both traditional Ryukyuan citrus and citrus imported from other locations, as well as a few additional accessions from mainland Japan for comparative purposes.

Additional native Ryukyuan citrus

- Oto (OTO), Kabuchii (KBU) and Tarogayo (TRG) were obtained from Asahikawa, Nago City, Okinawa, provided by Hirofumi Higa. These are traditional Okinawan citrus that we found to be half-siblings and part of a larger family as described in **Supplementary Note 6**.
- We sampled two citrus described as 'yukunibu' ('sour citrus' in Okinawan dialect):
  - R03 was obtained from Hama, Kunigami Village, Okinawa, provided by Chogi Miyagi.
  - R19 was obtained from Katsuyama, Nago City, Okinawa, provided by Hideki Yamashiro.
- Unzoki (R04) was obtained from Nuha, Oogimi Village, Okinawa, provided by Kiyoshi Miyagi.
- Keraji-mikan (KRJ) was obtained from Isagawa, Nago City, Okinawa, provided by Hideyasu Kinjo.
- Rokugatsu-mikan (RKG) was obtained from Isagawa, Nago City, Okinawa, provided by Hideyasu Kinjo.
- Deedee, or Ryukyu sour orange, (R00) was obtained from Tokijin, Nakijin Village, Okinawa, provided by Noboru Teruya.

Non-native Ryukyu citrus collected in Okinawa

- Kunenbo was collected from three different places. All three are confirmed to be clonally related by sequencing.
  - KB1 was obtained from Inamine, Nago City, Okinawa, provided by Tetsuo Shimabukuro.
  - KB2 was obtained from Oogimi, Oogimi Village, Okinawa, provided by Hiroshi Kobashigawa.

- KB3 was provided by a theme park called 'Okinawa World', managed by Nanto Co., Ltd. in Tamashiro, Nanjyo City, southern area of mainland Okinawa, through Tomohiro Chinen.

- Cleopatra (ISH) was obtained from Izumi, Motobu Town, Okinawa, provided by Eijyun Zamami.
- Shikinari-mikan (CAR), Bushukan (CI0 and CI1), pummelo × citron (CI2), Obeni (OBN), Tankan (TK0 and TK1) were provided by a theme park called 'Okinawa World', managed by Nanto Co., Ltd. in Tamashiro, Nanjyo City, Okinawa, through Tomohiro Chinen.

Other citrus collected in mainland Japan

- Yuzu (YZN) was obtained from Nikou, Nachikatsuura Town, Wakayama Prefecture, provided by Naoe Ooe.
- Yuzu (YZS) and daidai (DDS) were obtained from Shingu, Shingu City, Wakayama Prefecture, provided by Motofumi Yamasaki.

Other datasets

To augment our dataset with additional relevant sequences, we drew from previously published whole genome shotgun resequencing datasets[6-9]. These include a tachibana sample (M00) as noted above, and other accessions. A detailed list of these accessions can be found in **Supplementary Data 3**.

Variant calling and population genomic analysis

Illumina paired-end reads from each accession were mapped to the haploid Clementine reference sequence v1.0[7] using BWA-MEM[10]. Duplicate reads are removed with sambamba before calling variants with GATK HaplotypeCaller (version 3.7-0-gcfedb67)[11]. To reduce variant call errors, the following filters are applied as done previously[7]: variants are bi-allelic, read mapping quality >= 20, base quality >=20, genotype quality >=20, read depth between 1/2x′ and 2x the genome-wide median, allele balance on heterozygous SNPs in each sample using a binomial filter to exclude 5% of calls in the tails of the binomial distribution with probability 0.5 for sampling the alternative allele. To determine the chloroplast type of each accession, the Illumina paired-end reads are mapped to the chloroplast genome sequence of sweet orange[12] and variants are called using GATK as above.

For nuclear genome phylogenetic inference, we used single nucleotide polymorphisms in the introns and UTRs to minimize selection pressure bias. Each diploid genome was reduced to a haploid sequence by randomly sampling one allele at each variant position (the species phylogeny is insensitive to this sampling procedure). By requiring no missing genotype calls among the representative sequences, a total of 209,124 SNPs was obtained. A maximum likelihood tree was reconstructed with RAxML[1] under the general time-reversal model of nucleotide substitution with 1000 bootstrap replicates (‘raxmlHPC -m GTRGAMMA -N 1000’). The tree was rooted with Chinese box orange (*Severinia buxifolia*). The highly supported topology of the species tree (**Supplementary Fig. 2a**) is in agreement with our previously published nuclear genome phylogeny based on SNPs in complementary regions of the genome (non-genic, non-repetitive, non-pericentromeric)[3], affirming the robustness of the tree topology.

Supplementary Note 3. *Citrus ryukyuensis* sp. nov.

Multidimensional scaling, ancestry informative markers, and admixture

To analyze the population structure of east Asian citrus with a focus on Ryukyu citrus and mandarins, multi-dimensional scaling (MDS) analysis was performed using 51 citrus accessions including 3 pummelos, 20 mandarins, 2 oranges, 8 accessions of *C. ryukyuensis,* 3 tachibana, 6 shiikuwasha and 9 other indigenous Ryukyu hybrid types including what we learned are kunenbo × *C. ryukyuensis* hybrids and their derivatives, rokugatsu, and deedee (**Fig. 1a)**. A pairwise genome distance matrix was computed using the D metric[32] based on SNPs in genic regions before performing MDS with the cmdscale function from the R programing language.

For local ancestry inference and 4-way admixture analysis, we followed the procedure we previously used to assess other citrus species.[3] We derived ancestry informative marker SNPs (AIM) using representative accessions from each of the four populations (PU=pummelo, RK=*C. ryukyuensis*, MS=mangshanyeju, MA=common mandarins). The representative accessions are selected such that they contain the least admixture from other populations. As shown previously[32], this can be achieved by calculating pairwise distance D between accessions from two different populations in sliding windows. Representative accessions for three of the four populations included 3 pummelos, 3 accessions from *C. ryukyuensis*, and two accessions of mangshanyeju (MS1, MS2) (**Fig 1b**).

For the common mandarin population, pairwise D analysis showed that there are no pure accessions without admixture, and that the admixture pattern also varies among accessions and between chromosomes. We used Sun Chu Sha Kat (SCM) and Cleopatra (CLP) as exemplars for chromosomes 2-9 and Bendizao (M08) as exemplar for chromosome 1. Due to the weaker population differentiation between MS and MA relative to the other population pairs, we introduced a super population MM to denote segmental ancestry that is *C. reticulata* but finer distinction between MS and MA cannot be made due to the lack of ancestry informative SNPs at the subspecies level. This is analogous to human genome segmental ancestry inference where population ancestry assignment can be made at the continental but not regional level. Ancestry informative SNPs for MM are shared between MA and MS representative accessions.

We obtained a total of 397,887 AIMs: 268,383 for PU, 54,325 for RK, 35,067 for MA, 22,482 for MM, and 17,630 for MS. Sliding windows of 500 AIMs were used for local ancestry inference.

For relatedness analysis, haplotype sharing between accession pairs were calculated in sliding windows of 200kb and coefficient of relatedness was estimated following Wu *et al.*[3]. For genomic regions exhibiting inter-specific hybrid ancestry, phased haplotypes instead of diploid genotypes were used for haplotype sharing calculation as done previously [32].

Whole genome admixture analysis was performed with ADMIXTURE[2] using genic SNPs. For Weir-Cockerham's Fst[33] calculation of population differentiation, we used vcftools[34] --weir-fst-pop.

Allele frequency spectrum of *C. ryukyuensis*

The folded allele frequency spectrum (AFS) for the 8 accessions of *C. ryukyuensis* from Okinawa was calculated by excluding genomic regions with *C. reticulata* admixture, and by restricting to sites where all 8 accessions have genotype calls (**Supplementary Fig. 3**). For demographic inference, we used moments[4] to model the folded AFS to detect the possible existence of a population bottleneck. We compared the log-likelihoods of a panmictic constant population size model (no bottleneck) and a two-epoch mode characterized by an ancestral effective population size N_a and a current population size N_b that persists till T generations ago. Convergence of the simulation in terms of the log-likelihood value was checked by at least five runs with different, random starting values of the model parameters.

Due to the small number of *C. ryukyuensis* samples, the AFS cannot be used to uniquely determine the population sizes and time duration of the bottleneck. Rather, equally good fits (in terms of log-likelihood) are found as long as $0.1 < N_b/N_a < 0.7$, with corresponding estimates for $T/(2*N_a)$. In other words, the AFS is only good for finding a valley in the landscape of the 2-epoch model's parameter space. Compared to the constant population size model (log-likelihood=-423.891), the two-epoch bottleneck model (log-likelihood=-377.928 for $0.1 < N_b/N_a < 0.7$) provides a better fit.

The statistical significance of the bottleneck can be assessed using the likelihood ratio test, with the constant population size model as a special case of the two-epoch model (i.e., $N_b=N_a$). By comparing the test statistic (-2*(-423.891 - (-377.928))=91.93) to a chi-square distribution with two degrees of freedom, the *P*-value is estimated to be 1.1×10^{-20}, ruling out the constant population size model in favor of a population bottleneck for describing the Okinawa *C. ryukyuensis* population. Though the bottleneck intensity cannot be estimated from the single population AFS due to the small sample size, it can be estimated in a four-population model describing the East Asian citrus speciation (**Supplementary Note 11**).

The inferred population bottleneck is consistent with the much lower nucleotide diversity of *C. ryukyuensis* than the mainland Asian mandarins (**Fig. 1c**). The eight *C. ryukyuensis* accessions from Okinawa form a genetic cluster distinct from tachibana, shiikuwasha and other citrus types in multidimensional scaling analysis (**Fig. 1a**). Unlike tachibana and shiikuwasha, they are monoembryonic (**Supplementary Fig. 1**). The AFS is consistent with *C. ryukyuensis* of Okinawa being a panmictic population.

Phenotypic differences between *C. ryukyuensis*, tachibana, and shiikuwasha

In order to describe the morphological characteristics for flowers and fruits, including seeds, samples were generally harvested from the same tree from which we collected the leaves for sequencing, if there were reproductive organs. In cases where this was not possible, flower and fruit samples were collected from different trees of the same variety based on name and/or genotype.

Compared with tachibana and shiikuwasha, *C. ryukyuensis* has the smallest fruit size. Although the fruit size of shiikuwasha is highly variable, shiikuwasha fruit are typically larger than the fruit of tachibana and *C. ryukyuensis*. While shiikuwasha fruit has a characteristically depressed or flat shape (giving rise to the species designation *C. depressa*), tachibana has the most rounded

fruit. The fruit skin color of tachibana, shiikuwasha and *C. ryukyuensis* at the time of observation was light yellow, yellow-orange, and yellow, respectively. The color of fruit pulp of tachibana, shiikuwasha and *C. ryukyuensis* is light luteofulvous, yellow, and light yellow, respectively. The shiikuwasha leaf size is bigger than that of tachibana and *C. ryukyuensis*. The shape of the leaf blade of *C. ryukyuensis* is narrower than that of tachibana and shiikuwasha.

Relationship of *C. ryukyuensis* to Tanaka's 'tanibuta'

In his survey of Ryukyu citrus[12], Tanaka described a 'tanibuta' accession that he referred to as 'Ryukyu tachibana', and assigned it the name *C. tachibana* Tanaka *var. attenuata* in his classification system. Tanaka noted that unlike tachibana from mainland Japan, which are polyembryonic, the 'tanibuta' he examined was monoembryonic. As noted above (**Supplementary Note 1**), the genetic difference between 'tanibuta' from Okinawa and tachibana from the main islands of Japan has previously been discussed by Yamamoto based on molecular marker analysis.[7]

Tanaka's description of tanibuta[12] mostly matches the morphological characteristics of our *C. ryukyuensis* accessions. The tanibuta accession TBR that we sequenced clearly belongs to the *C. ryukyuensis* cluster and, except for a short insertion of *C. reticulata*, is a pure *C. ryukyuensis* (**Fig. 3b**). This clearly differentiates it from mainland tachibanas, which are predominantly *C. ryukyuensis*/*C. reticulata* hybrids (**Fig. 3b**, **Supplementary Note 5**). As with Tanaka's 'tanibuta' accession, ours are monoembryonic, another key difference from mainland tachibana.

Tanaka, however, suspected that the 'tanibuta' he examined was a natural tetraploid, although he did not provide details. Our named tanibuta accession TBR (and other members of our *C. ryukyuensis* clade) lack the typical features of tetraploid citrus, which compared with diploids (1) are generally smaller trees, (2) have broader and rounder leaf blades, (3) are generally darker in color, (4) have fewer but larger oil glands on leaves, leading to a rougher texture, and (5) have coarser fruit surfaces. Due to these possible phenotypic differences between our 'tanibuta' accessions, the 'tanibuta' described by Tanaka, and mainland Japanese tachibana, to avoid confusion we do not refer to them as Ryukyu tachibana. As shown in **Supplementary Notes 4 and 5**, *C. ryukyuensis* is (1) a distinct sexual species, and (2) ancestral to both shiikuwasha and tachibana.

Supplementary Note 4. Nature of shiikuwasha (*C. depressa* Hayata)

Identification of numerous clones of shiikuwasha

Shiikuwasha (*C. depressa* Hayata) are indigenous to the Ryukyu Islands. They have been suspected to be of hybrid origin though the parents were not known previously.[7,13] Genome comparison of the 29 accessions of shiikuwasha in our collection shows that they are derived from six distinct genomes each with varying numbers of clonal relatives (**Supplementary Data 1**). The most common type is Kuganii shiikuwasha with 15 clonal relatives. As previously observed[7], there are two distinct chloroplast types among the shiikuwasha. Four of the six basic genotypes (including kuganii=SH3 and njakuganii=SH0 types) have *C. ryukyuensis* cpDNA and the other two (*i.e*., kaabishii=SH1, kachii=SH2) have *C. reticulata* cpDNA (**Supplementary Fig. 2b**). The shiikuwasha type (SHF) from the USDA National Clonal Germplasm Repository for Citrus is a clonal relative of kaabishii (SH1). This accession (SHF) was a gift by T. Tanaka to Swingle[2]. Shiikuwashas are apomictic which in turn facilitated their clonal expansion.

Distinct shiikuwasha types are half-sibs sharing the same extant mainland Asian mandarin parent

Genome wide local ancestry analysis shows that different shiikuwasha types are interspecific hybrids derived from *C. ryukyuensis* and *C. reticulata* (**Fig. 3b**). We note also the presence of one pummelo segment (~ 2Mb) near the beginning of chromosome 3 in five of the six basic genotypes (**Fig. 3b**), as found in Cleopatra and Sunki mandarins, indicating type-2 mandarin ancestry with little pummelo admixture[3]. To understand the genetic relatedness among the six basic shiikuwasha types, genome wide interspecific phasing was performed using representative *C. ryukyuensis* and mainland Asian mandarins to define alleles for the two parental gene pools respectively, following Wu *et al.*[3] Haplotype sharing analysis based on the phased shiikuwasha genomes shows that the six basic genotypes have distinct *C. ryukyuensis* parents but share the same mainland mandarin parent. As both *C. ryukyuensis* and *C. reticulata* cpDNA types are observed among the six basic types, this mandarin parent was inferred to be the seed or pollen parent during different hybridization events at the origin of shiikuwasha, consistent with previous observations[7].

Remarkably, we found that the singular mandarin parent of all the sequenced shiikuwasha has the same genotype as a mandarin we collected from the garden of the Katsuyama Shiikuwasha Co. Ltd. in Nago City, Okinawa (RK3). This Katsuyama ishikunibu (1) shares the *C. reticulata*-type cpDNA of two of the six shiikuwashas genotypes (SH1, SH2), and (2) has genome-wide haplotype sharing with each shiikuwasha, as expected if RK3 were the common parent of all shiikuwasha. RK3 exhibits the nucellar embryony phenotype and can therefore reproduce asexually by seed (see **Supplementary Note 10**). Thus, we conclude that Katsuyama ishikunibu (RK3) is clonally related to the mainland Asian mandarin parent of all shiikuwashas.

The 1948 book[35] of Y. Tanaka contains a drawing of a Ryukyuan citrus he called 'ishikunibu'. While the drawing conveys limited morphological characteristics, among our collection 'Katsuyama ishikunibu' (RK3) is the closest. By comparing RK3 to all the sequenced mandarin

genomes, the closest relative is found to be Sun Chu Sha Kat (SCM), a pure (without interspecific admixture) ancient Chinese mandarin with coefficient of relatedness 0.41. RK3 is also related to Cleopatra (ISH) and sunki (SNK).

Origin of Nakamoto seedless shiikuwasha

Our collection included one seedless shiikuwasha (Nakamoto seedless[36], SH4) provided by the Okinawa Prefectural Agricultural Research Center. Compared with other shiikuwasha exemplified by the popular kuganii variety, the tree vigor of Nakamoto seedless is weaker, fruit enlargement is slower and ultimate fruit size is smaller. The seedless fruit ripens in advance of seeded shiikuwasha, with early reduction of acid, and is less aromatic. The rind of seedless shiikuwasha is thicker than kuganii. The underlying shape of the fruit's multiple carpels is also evident, arising from incomplete symphysis of the carpels.

Comparative genome analysis shows that seedless Nakamoto shiikuwasha (SH4) is related to the kachii shiikuwasha (SH2) by somatic mutations in the form of long runs of homozygosity (*i.e.*, loss of heterozygosity) on chromosomes 2 and 5 of the seedless shiikuwasha (**Fig. 3b**). More specifically, most of chromosome 2 (31.6 of 36.4 Mb) of the seedless shiikuwasha is homozygous for a *C. ryukyuensis* haplotype, whereas the kachii shiikuwasha contains the same *C. ryukyuensis* haplotype paired with a *C. reticulata* haplotype. Likewise, most of chromosome 5 (37.5 of 43.3 Mb) of the seedless shiikuwasha consists of two identical *C. reticulata* haplotypes, whereas the same region of the kachii shiikuwasha is heterozygous for the same *C. reticulata* haplotype and a *C. ryukyuensis* haplotype. The rest of the seedless shiikuwasha genome is highly heterozygous and shares both haplotypes with the kachii shiikuwasha. The loss of heterozygosity on chromosomes 2 and 5 is not due to segmental loss, since the depth of coverage remains the same. Such a condition can arise from mitotic recombination, which results in terminal homozygosity[37,38]. Long runs of homozygosity have also been observed in other plant genomes such as pineapple[39], and may have played an important role in plant domestication.

These observations are compatible with the idea that the seedless phenotype of Nakamoto shiikuwasha could be a consequence of these chromosomal changes that cause meiotic defects and promoting seedlessness. Alternately the seedless trait could be controlled by one or more genes in the homozygous regions of chromosomes 2 and/or 5.

Supplementary Note 5. Nature of tachibana (*C. tachibana* (Makino))

Three distinct tachibana genotypes

We have genome sequences of nine tachibana accessions in our collection (**Supplementary Data 1**), including 2 from previous publications (M00 from [31], TBM from [3], **Supplementary Data 3**). All nine accessions have *C. ryukyuensis* cpDNA. Comparative genome analysis shows that these nine accessions are clonally derived from three basic genotypes each with its own characteristic admixture pattern (**Fig. 3b**). One genotype (mainland tachibana) contains 6 accessions that are clonally related (TB0, TB3, TB4, TB6, TB7, TBM). A second genotype (Nikou tachibana) includes 2 clonal relatives TB5 and TB8; the third genotype is represented by tachibana accession M00.

All nine tachibana accessions are heterozygous for the polyembryonic allele **(Supplementary Note 10)** of *CitRKD1* [31,40]. Phenotypically, they are all polyembryonic and therefore have undergone predominantly asexual propagation. This is consistent with the observed abundance of clonal relatives in tachibana populations[16,19].

Complex admixture patterns in tachibana

Whereas shiikuwasha originated from interspecific hybridizations of a clonal relative of the Katsuyama ishikunibu (RK3) with different *C. ryukyuensis* individuals followed by clonal propagation, tachibana genomes display a mosaic admixture pattern (**Fig. 3b**). Based on regions where local ancestry can be confidently assigned, all three tachibana genotypes are characterized predominantly by interspecific (*C. ryukyuensis/C. reticulata*) admixture (TB0: 94%, TB5: 80%, M00: 93%). However, all three genomes also have 5-8 segments of *C. ryukyuensis*/*C. ryukyuensis* ancestry (TB0: 6.3%, TB5: 20%, M00: 6.9%). This indicates that the three tachibanas originated from hybridizations between *C. ryukyuensis* and admixed individuals of *C. reticulata* with approximately 6-20% *C. ryukyuensis* ancestry. Indeed, a previous study suggested the existence of hybrid populations between mandarin (*C. reticulata*) and tachibana[16].
The interspecific admixture patterns in different tachibana types are in line with the observed genotype diversity of three isozyme genes among 113 tachibana trees representing four distinct genotypes[16]. Unlike shiikuwasha, no pummelo admixture is found in tachibana. Our result on the hybrid ancestry of tachibana, derived solely from *C. ryukyuensis* and *C. reticulata*, differs from earlier proposals that tachibana are mandarin-citron hybrids[19] or hybrid genotypes with *Fortunella* introgression[41].
The interspecific admixture of tachibana called for a revision of the taxonomical systems of Swingle and Tanaka in which tachibana was considered a pure species.

Relatedness of the three tachibana genotypes

Pairwise haplotype sharing between the three distinct tachibana genotypes is calculated after phasing the interspecific (*C. ryukyuensis/C. reticulata*) hybrid regions of each genome, following[3]. Significantly more haplotype sharing is observed from the *C. reticulata* ancestry than from the *C. ryukyuensis* ancestry (**Supplementary Fig. 6b**). The lack of haplotype sharing from the *C. ryukyuensis* ancestry suggests that independent hybridization events involving different *C. ryukyuensis* individuals may be at the origin of the three distinct tachibana genotypes described here, similar to the formation of the six shiikuwasha genotypes.
Further insight into the origin of tachibana can be obtained by comparing the *C. reticulata* haplotypes in genomic regions where all three tachibana genotypes are hybrid *C. ryukyuensis/C.*

reticulata. Local haplotype trees in sliding windows of 1Mb show that at most two distinct *C. reticulata* haplotypes are in the tachibana genomes. By contrast, the three genotypes of tachibana carry three distinct *C. ryukyuensis* haplotypes (**Supplementary Fig. 6a**). This suggests that 1) only one mainland Asian mandarin (*C. reticulata*) ancestor contributed to the gene pool of tachibana, and 2) the three tachibanas have different *C. ryukyuensis* parents. These results are at variance with a previously proposed model where tachibanas are full siblings sharing two parents[19].

Significant mangshanyeju admixture in tachibana but not in shiikuwasha

Four-way admixture analysis (**Figs. 1b** and **3b**) further shows that there are comparable mangshanyeju (MS) and common mandarin (MA) contributions to the genetic makeup of the three tachibanas (MS =18-22%, MA=19-20%). This suggests that the Chinese migrant mandarin from which tachibana derives its *C. reticulata* ancestry is likely a hybrid between the two subspecies of *C. reticulata* (MS and MA). This unknown mandarin ancestor is inferred to carry the polyembryonic allele with MITE haplotype H1A which is inherited by all three tachibana types. By contrast, the shiikuwasha genomes have little MS ancestry (3.4-6.8%) relative to the common mandarin ancestry (MA=37-39%), and all sequenced shiikuwashas inherit the polyembryonic allele with MITE haplotype H1B from their mandarin parent (RK3).

An evolutionary model for tachibana

Based on the above observations, a plausible model for the sequence of events leading to the origin of tachibana can be proposed. It started with one apomictic Chinese mandarin ancestor which is itself a hybrid between two *C. reticulata* populations, *i.e.*, mangshanyeju and common mandarins. This mandarin ancestor migrated to the Japanese main islands after the establishment of a *C. ryukyuensis* population there. A clonal population of this mandarin ancestor hybridized with the local *C. ryukyuensis* population, followed by complex backcrosses resulting in admixed mandarins with varying degree of *C. ryukyuensis* ancestry. Some admixed mandarins carrying the polyembryonic allele of *CitRKD1* underwent further clonal expansion and hybridization with the *C. ryukyuensis* population, resulting in tachibana.

It is not clear whether this mandarin ancestor also arrived to Taiwan and the Ryukyu Islands. Tanaka reported the presence of tachibana in Taiwan, the Ryukyu Islands and mainland Japan[42]. However, as noted in **Supplementary Note 3**, the so-called Ryukyu tachibana (*Citrus tachibana* Tanaka var. *attenuata*, aka tanibuta in Okinawa dialect) was reported to be monoembryonic[12] unlike tachibana from mainland Japan, and sequence analysis shows that the tanibuta accessions in our collection represent a new species *C. ryukyuensis* and are monoembryonic with little admixture from *C. reticulata* (**Fig. 3b**). Future sampling and sequencing effort especially from Taiwan will provide a more detailed picture about the diversity and evolutionary origin of tachibana and other citrus types.

Supplementary Note 6. Yukunibu group: Kunenbo×*C. ryukyuensis* hybrids and relatives

A half-sib family from the Ryukyu Islands

By genome wide interspecific phasing and haplotype sharing analysis, we found that several Ryukyuan citrus types are related to each other as half-sibs with the kunenbo mandarin as their common seed parent and with distinct pollen parents belonging to *C. ryukyuensis* (**Fig. 3a; Supplementary Fig. 7**). Later in this section, we propose that this group be designated *C.* × *yukunibu*. The specific name yukunibu means 'sour citrus' in Okinawan dialect, and it has been used as a colloquial descriptor for several of our samples.

Three members of this family are half-sibs that have been classically referred to as different species[43]: oto (*C. oto* hort. ex Yu. Tanaka), kabuchii (*C. keraji* var. *kabuchii* hort. ex Tanaka), and tarogayo (*C. tarogayo* hort. ex Tanaka). The parent-child relationship between kunenbo and kabuchii was previously noted[19].

A fourth type related to kunenbo, keraji-mikan, is a backcross of kabuchii to kunenbo as previously noted.[19] and now confirmed by our genomic analysis. Since both have the same cpDNA, we cannot determine which is the maternal parent. Keraji-mikan has also been assigned taxonomically to *C. keraji*, although Yamamoto and colleagues recognized that kabuchii and keraji-mikan are distinct both phenotypically and with molecular markers[44]. Yamamoto *et al.* differentiate between keraji-type, a nearly seedless type with smooth rind, and 'kabuchii-type' that is seedy with rough rind texture.

Through sequence analysis we identified two additional members of the oto-kabuchii-tarogayo-keraji family, both colloquially designated yukunibu. These are (1) 'yukunibu (Nuha)' R04, also known as unzoki and (2) an unnamed yukunibu represented in our collection by two clones, R03 and R19. While unzoki (R04) has rough skin, yukunibu (R03/R19) has smoother skin.

Finally, we find that an accession in our collection (Kaneshi, R07), originally labeled as a shiikuwasha, is a cross between kabuchii and Njakuganii shiikuwasha (SH0 or clonal relative), with kabuchii as the seed parent.

The seven members of this family inherited the polyembryonic allele from their kunenbo seed parent (itself being heterozygous for this allele), with keraji-mikan being homozygous for this allele as a result of the backcross. All seven accessions are inferred to be polyembryonic and inherit the sweet orange cpDNA type from kunenbo. The existence of a family of recent hybrids with kunenbo is consistent with historical records that show the importation of kunenbo to the Ryukyu Islands from Indochina prior to 1564[45] or during the 8th century [19] (For more on kunenbo and its relationships, see **Supplementary Note 8**.)

Suggested nomenclature

Citrus taxonomy and nomenclature are evolving to take into account information from genome analyses[46]. While genomics can definitively identify relationships, taxonomic nomenclature aims to provide universally understood terminology to refer to groups of accessions, and to signal higher order relationships. This is complicated in citrus and other groups by the prevalence and variety of interspecific hybrids. According to formal nomenclatural principles, all hybrids with the same input species are generally assigned the same name. By this principle, all hybrids of pummelo and mandarin can be referred to as *C.* × *aurantium*; this approach, however, groups disparate accessions like sweet and sour oranges into the same taxon. Another drawback to conventional taxonomic nomenclature is that it does not have a natural way to represent full or half-sib families.

Members of the half-sib family derived from natural hybridization of kunenbo with diverse accessions of *C. ryukyuensis* have previously been assigned individual species names that obscure the overall relatedness of the group, including *C. oto* (oto), *C. tarogayo* (tarogayo) and *C. keraji*. This latter name has been used for both kabuchii, as *C. keraji* var *kabuchii* (T. Tanaka), and keraji-mikan known from Kikajima Island in Kagoshima Prefecture.[44] As noted above, keraji-mikan is a backcross of kabuchii with kunenbo.[19] In order to indicate the hybrid nature and familial relationship among these varieties, we propose to refer to this extended family as the 'yukunibu group', from the word 'yukunibu' meaning 'sour citrus' in Okinawan dialect, and suggest *C.* × *yukunibu* for a formal name, with specific type names as (clonal) varieties, e.g., *C.* × *yukunibu* var. *oto,* etc.

Supplementary Note 7. Other citrus with Ryukyuan ancestry

The Ryukyu sour orange deedee

The Ryukyu sour orange, deedee, is a cultivar indigenous to the Ryukyu islands that is distinct from daidai which, as noted in **Supplementary Note 8**, is a clonal relative of sour orange (*C.* × *aurantium*) that was introduced to Japan. Our collection includes the cultivar Tokijin-Deedee (R00) from Nakijin Village with a pummelo cpDNA (**Supplementary Fig. 2b**). Local ancestry inference suggests it is a trispecific hybrid with ancestry composition of 50% pummelo, 21% *C. ryukyuensis* and 28% *C. reticulata* (remaining 1% cannot be assigned) (**Fig. 1b**). The admixture pattern (**Fig. 3b**) suggests that it originated from a cross between a pummelo seed parent and a hybrid pollen parent with both *C. ryukyuensis* and *C. reticulata* ancestry.

Further insight into the parentage of deedee is obtained with interspecific phasing and haplotype sharing analysis. The *C. reticulata* haplotype matches the singular mandarin parent of the shiikuwasha (i.e. RK3), whereas the *C. ryukyuensis* haplotype is distinct from that of the six basic shiikuwasha genotypes. The pollen parent of deedee is thus inferred to be a shiikuwasha not found in our sequenced shiikuwasha accessions.

The Ryukyu sour orange (deedee) is heterozygous for the polyembryonic allele that can be traced to its grandparental genotype RK3 through its shiikuwasha pollen parent.

Rokugatsu-mikan (aka zadaidai sour orange)

Rokugatsu-mikan (*Citrus rokugatsu* hort. ex Yu.Tanaka , aka zadaidai sour orange) is a well-known variety in Japan and indigenous to the Ryukyu islands[12,47]. We sequenced one accession of rokugatsu-mikan (RKG), and it has a *C. ryukyuensis* cpDNA (**Supplementary Fig. 2b**). Phasing and haplotype sharing analysis shows that it originated as an F1 hybrid *C. ryukyuensis* × daidai. Rokugatsu is polyembryonic and heterozygous for the polyembryonic allele of the *CitRKD1* gene[40].

Regarding the parentage of rokugatsu, Shimizu *et al.*[19] also found that daidai was a parent of rokugatsu (with the other parent undetermined) but they cite daidai as the seed parent, in contrast to cpDNA analysis of our accession; it is possible that there is more than one genotype called rokugatsu.

Supplementary Note 8. Additional relationships among Japanese and Chinese citrus

In our broad sampling of citrus grown in the Ryukyus, we also sequenced several additional cultivars that did not have any *C. ryukyuensis* ancestry. Because of the commercial importance of these cultivars, we summarize our findings here.

Kunenbo and kishu mandarins

Kunenbo-mikan (*Citrus nobilis* Lour. var. *kunip* Tanaka) is a particularly important type in the history of Japanese citrus[19,45]. As described in the main text and **Supplementary Note 6**, we found that kunenbo is the seed parent of the yukunibu-group of Ryukyu citrus, with pollen parents drawn from the native *C. ryukyuensis* population.

We sequenced the genome of kunenbo from three Ryukyuan trees (KB1, KB2, KB3, **Supplementary Data 1**), and found them to be clones. Molecular marker-based studies[19,48] shows that kunenbo is a clonal relative of the Chinese mandarin 'bendiguangju' that is not in our collection. Although many other cultivated mandarins show modest pummelo admixture[3], the pummelo content of Kunenbo-mikan (37%) is comparable to sweet orange (45%), and the two share the same cpDNA as previously noted[19].

We confirm that kunenbo itself is the offspring of kishu mandarin (*Citrus kinokuni* hort. ex Tanaka), with an unknown seed parent, in agreement with previous analyses based on limited molecular markers[19]. Kishu is monoembryonic[40] and does not carry the polyembryonic allele of *CitRKD1*. By contrast, kunenbo is polyembryonic and heterozygous for the polyembryonic allele of *CitRKD1*. Together with the pummelo admixture patterns in kunenbo and kishu, this implies that the seed parent of kunenbo has mostly pummelo ancestry but with *C. reticulata* admixture carrying the polyembryonic allele of *CitRKD1*. Based on genome sequence comparison we confirm that mukaku-kishu (seedless kishu, from Riverside collection[3]) is clonally related to the to the recently published Nanfengmiju[49], in agreement with molecular marker based analysis[19].

Since kunenbo is the seed parent of the yukunibu group, this family must have arisen after the introduction of kunenbo to the Ryukyu islands. The precise timing of this introduction is unclear[19,50]. Kajiura[51] wrote that kunenbo was introduced to the Ryukyus and then mainland Japan from Indochina circa 1564.[45,51] Tanaka[52,53], however, believed in an earlier introduction (the end of 11th to the end of 12th century). Some authors have identified kunenbo with other citrus and inferred an earlier introduction. Kajiura took kunenbo to be synonymous with 'abetachibana'. According to the 'Honzo Wamyou', the oldest medicinal substance dictionary, this would imply introduction by 918. Finally, while Kajibara [1709] identified kunenbo with ‘koji', which was introduced to Japan in 725, but the equivalence of kunenbo and koji is not widely accepted[19]. In any event, kunenbo was clearly introduced to the Ryukyu Islands no later than 1564.

Finally, we note in passing that although King mandarin (*C. nobilis* Lour) and kunenbo (*Citrus nobilis* Lour. var. *kunip* Tanaka) are both given the name *C. nobilis* in the Tanaka system, they are not closely related. Further, neither King nor kunenbo is a tangor (with sweet orange as direct parent).

Satsuma mandarin and its relationships

Satsuma (in the Tanaka system, *C. unshiu*, reflecting its putative ties to Wenzhou, China) is the most commonly grown citrus in Japan, and was already present there by 1648[50]. By whole genome analysis we confirm that the popular satsuma mandarin is an F1 hybrid of kishu mandarin and kunenbo, with kunenbo as the pollen parent, as previously hypothesized based on a limited number of markers[19,48] and by an elegant analysis of self-incompatibility loci[45]. Since kunenbo is the offspring of kishu mandarin as noted above, satsuma is a first-generation backcross. Satsuma mandarin is heterozygous for the polyembryonic allele of *CitRKD1* carrying the MITE H1A haplotype (**Supplementary Note 10**).

Tankan-mikan

Tankan-mikan, a medium-sized, subglobose fruit produced on a tree with typical mandarin growth habit, has been considered to be a tangor,[19] with sweet orange as its pollen parent. We sequenced two different trees, TK1 and TK0, which were clonally related. We confirmed that Tankan is an F1 hybrid of an unknown mandarin and sweet orange, in line with previous marker-based observation[19]. Tankan is heterozygous for the nucellar embryony allele of *CitRKD1* and carries the MITE haplotype H1B from its unknown mandarin parent.

Obeni-mikan is clonally related to Dancy

We find that Obeni-mikan (OBN; also written as 'oobeni'), another admixed mandarin, is clonally related to Dancy thereby confirming results from single nucleotide polymorphism array analysis[48]. Dancy was previously inferred to be a child of ponkan mandarin based on whole genome sequence comparison[3].

Yuzu (*C. junos* Sieb. ex Tanaka) is an F1 hybrid of Ichang papeda × Mangshanyeju

Yuzu (*C. junos* Sieb. ex Tanaka) originated in China and was introduced to Japan via Korea before the eighth century[54]. Besides being used in food products, the appealing aroma of yuzu has been prized for mind-body health in Japan.[55] It is thought to have resulted from an accidental cross-pollination of some cultivated variety of the mandarin orange by the Ichang papeda[13].

We collected two accessions of yuzu, Nikou yuzu (YZN) and Shingu yuzu (YZS) from mainland Japan. Sequence comparison shows that the two accessions are clonally related with a *C. ichangensis* cpDNA (**Supplementary Fig. 2b**). Admixture analysis confirms that yuzu is an interspecific hybrid of *C. ichangensis* × *C. reticulata* and further reveals that the *C. reticulata* parent was a mangshanyeju (MS) wild mandarin (**Supplementary Fig. 4a**). Interestingly, yuzu is the only known interspecific hybrid with a pure MS parent. By comparison, the other sequenced mandarin hybrids (sour orange, calamondin, Rangpur lime, rough lemon and shiikuwasha) derive their mandarin (*C. reticulata*) ancestry mostly from the common mandarin population (**Supplementary Fig. 4a**) with the exception of tachibana, whose Chinese mandarin ancestor was a hybrid between mangshanyeju and common mandarin (**Supplementary Note 5**).

Our yuzu accessions exhibit nucellar embryony and are heterozygous for the polyembryonic allele of *CitRKD1*. In particular, the MITE DNA transposon sequence associated with the polyembryonic allele represents a unique haplotype (MITE H2B, **Fig. 4a**) that is not found in other sequenced mandarins or mandarin hybrids. ADMIXTURE[2] analysis at the apomixis locus shows that the polyembryonic allele in yuzu originated from the mangshanyeju population (**Fig. 4b, Supplementary Note 10**).

Yuzu's unique mangshanyeju ancestry might be explained by the fact that wild Ichang papeda are found grown in sympatry with mangshanyeju[49]. This would imply Mangshan region as the original birthplace of yuzu. Presumably, the inherited apomixis trait from mangshanyeju and cold-hardiness trait from Ichang papeda allow the clonal propagation of yuzu into much wider geographical areas and variable climates.

Bushukan (*C. medica* var. *sarcodactylis*)

Citrons (*C. medica*) are widely grown in India and Southwest China with great phenotypic[56] and genetic diversity[57,58]. We sequenced two different bushukan (meaning 'Buddha's Hand', *C. medica* var. *sarcodactylis*) from Okinawa World Theme Park, CI0 and CI1. Sequence comparison shows that they are clonal relatives and have parent/child relationship with the previously sequenced[3] 'Buddha's hand' citron (BUD). All three have the same cpDNA.

Shikinari-calamansi is clonally related to calamondin

Our sequenced shikinari-calamansi (CAR) is clonally related to the previously sequenced[3] calamondin (CAL) also known as Philippine lime or *C.* × *microcarpa*. Calamondin is a hybrid of kumquat and mandarin[3]. (Shikinari means 'four seasons'.)

A pummelo × citron hybrid

We sequenced an accession (denoted CI2 in our collection) identified as a citron from 'Okinawa World Theme Park' (Tamashiro, Nanjyo city, Okinawa). This accession is a novel pummelo × citron hybrid (with pummelo as the seed parent). It is therefore a member of the same nothospecies *C.* × *lumia* Risso & Poit., which includes varieties with the common name pear lemon, French lime, and sweet lemon[59].

Daidai (*C. aurantium*) is clonally related to Seville sour orange

The daidai accession reported here was collected from mainland Japan. Sequence comparison shows that daidai (DDS) is a clonal relative of the previously sequenced[3] Seville sour orange (*C.* × *aurantium*) and originated as an F1 hybrid of *C. maxima* × *C. reticulata* (**Fig. 4c**). Daidai was introduced to Japan around 61 CE[45].

Supplementary Note 9. Mangshanyeju and common mandarins

Mangshanyeju and common mandarin are distinct populations

As described in the main text, wild mandarins from Mangshan in southern Hunan province of China, known as 'mangshanyeju' (MS), are a population or sub-species of *C. reticulata* distinct from 'common mandarin' (MA). We use 'common mandarin' in two different contexts. First, as a subspecies of *C. reticulata*, MA refers to a genetically pure population that is strongly differentiated from MS. Second, 'common mandarin' can also refer to a particular mandarin type whose genetic ancestry is predominately of the subspecies MA with possible admixture from MS and/or pummelo. In the latter sense, 'common mandarins' include all the commercially valuable varieties, both domesticated (in the sense of low acid, such as Clementine and satsuma) and acidic types (e.g., Cleopatra).

Three lines of evidence support genome-wide population differentiation between common mandarins and mangshanyeju:

1) multi-dimensional scaling (MDS) analysis,
2) sequence divergence between MS and MA, and Fst
3) admixture analysis

MDS analysis of 51 citrus accessions (excluding clonal relatives) including pummelos (*C. maxima*), mandarins (*C. reticulata*), oranges and accessions of *C. ryukyuensis,* tachibana, shiikuwasha, and other indigenous Ryukyu types, reveals the population structure at progressively finer detail. The first two principal coordinates separate the three species of *C. maxima* (pummelos), *C. reticulata* (mandarins) and *C. ryukyuensis* with admixed and hybrid accessions at intermediate positions of their ancestral species (**Fig. 1a**). The third principal coordinate separates out the two wild Mangshan mandarins (MS1 and MS2; together, MS), with two other wild mandarins (M01=Chongyi wild mandarin, and M04=Suanpangan) at an intermediate position between MS and common mandarins. This indicates that MS represents a distinct population from common mandarins, suggesting that some extant wild mandarins resulted from hybridization between the two populations.

As a second line of evidence, we examined the pairwise fixed differences (AA|BB, or 'identical by state' IBS0) relative to shared heterozygous SNPs (AB|AB or IBS2), where A and B denotes two distinct alleles. Between a common mandarin (SCM) and the MS accession MS1, IBS0/IBS2 =3.73. By comparison, IBS0/IBS2= 0.16 between two common mandarins CLP and SCM. The excess of fixed differences (IBS0) indicates population differentiation between MS and common mandarins. (It can be shown[60] that for a panmictic population, IBS0/IBS2=0.5 for a pair of unrelated diploids. Population differentiation leads to higher values for IBS0/IBS2, whereas relatedness decreases IBS0/IBS2). Using CLP and SCM to represent common mandarins and MS1/MS2 to represent MS, Weir-Cockerham's[33] Fst is estimated to be 0.49 after excluding genomic regions with MS admixture in CLP and SCM. Phylogenetic analysis also confirms the differentiation between mangshanyeju and common mandarins (**Supplementary Fig. 2a**).

The third line of evidence for considering mangshanyeju as a genetically distinct population and subspecies of *C. reticulata* is provided by admixture analysis. We first analyzed local ancestry

using ancestry-informative SNPs for the four ancestral populations inferred from MDS analysis (PU, RK, MS, MA) (**Fig. 1a, 1b**). It revealed wide-spread MS admixture in the common mandarins (**Fig. 1b**). As a complementary approach, population structure was analyzed using ADMIXTURE[2] based on 125,830 SNPs for 34 mainland Asian accessions by including three other ancestral citrus species (CI=citron, IC=Ichang papeda, FO=*Fortunella*) and interspecific mandarin hybrids but excluding accessions with *C. ryukyuensis* ancestry. Twenty runs were conducted for each K (number of ancestral populations) for K=2-8, and the run with the lowest cross validation error was used for each K. The ancestry compositions for K=4-6 ancestral populations are shown in **Supplementary Fig. 4a,** which reveals progressively finer resolution. At K=4, the two citrus species (IC, FO) are not resolved. All five citrus species are fully resolved at K=5, and further differentiation at the sub-species level between MS and MA is observed with six ancestral populations (K=6). In particular, yuzu (*C. junos* Sieb. ex Tanaka) is shown to be the only known interspecific hybrid involving MS as a direct parent, whereas common mandarin (MA) ancestry is at the origin of the other interspecific mandarin hybrids including sour orange (DDS), calamondin (CAR), Rangpur lime (LMA) and red rough lemon (RRL).
We conclude that mandarins (*C. reticulata*) are derived from two differentiated ancestral sub-populations that persist today. We estimate that these two sub-species diverged 1.4-1.7 million years ago (**Supplementary Note 11**). This early divergence suggests that both populations consist of wild mandarins throughout the Pleistocene epoch. More recently -- presumably sometime during the Holocene (11 kya-present) -- cultivated mandarins were selected in the common mandarin population. While MS population consists of wild mandarins from Mangshan, the MA population contains both wild and cultivated mandarins with varying degree of MS introgression. For instance, the two wild mandarin accessions M02 (Hezhou wild mandarin) and M03 (sour tangerine) have mostly MA ancestry with small amount of MS admixture. Genetically, they belong to common mandarins.

Widespread mangshanyeju introgression in common mandarins

Local ancestry analysis (**Fig. 1b**, **Fig. 4c**) shows that all sequenced common mandarins have varying degrees of MS admixture, especially on chromosome 1 (in the Clementine reference sequence coordinate system) where the *CitRKD1* gene regulating apomixis is located. Two wild mandarins (M01=Chongyi wild mandarin, M04=Suanpangan) are hybrid between MS and MA (see also **Supplementary Fig. 4a**). Genome comparison shows that M01 is clonally related to 4 other previously sequenced[49] wild mandarins (DX2, DX3, DX4, JYY) from southern Hunan province. The geographic distribution of this hybrid wild mandarin type could have been facilitated by asexual reproduction via by nucellar embryony.

Note from **Supplementary Fig. 4a** that the whole genome ancestry composition inference software ADMIXTURE[2] fails to detect the small amount of pummelo admixture in type 2 mandarins[3] except M08 (Bendizao). Similarly, ADMIXTURE is not sensitive to small amount of MS admixture in some common mandarins as identified by local ancestry inference (**Fig. 1b**).

Supplementary Note 10. Diversity and origin of apomixis alleles, and the loss of anthocyanin biosynthesis

Diversity of apomixis alleles

Apomixis (asexual reproduction via seed) has played an important role in citrus propagation and particularly in domestication, allowing desirable genotypes to be immediately fixed and propagated. Apomixis in citrus is the result of nucellar embryony, which often (but not always) generates multiple somatic embryos per seed. For this reason, citrus researchers frequently use the term 'polyembryony' interchangeably with nucellar embryony, although they are not formally identical phenomena. In this sense, polyembryony is dominantly inherited in citrus[8]. A major locus for polyembryony was initially identified[61] spanning 380 kb containing 70 open reading frames. More recently, the locus has been narrowed down to an 80kb region containing 11 candidate genes[31]. Of these, the *CitRWP*[31] gene (a.k.a. *CitRKD1*[40]) was correlated with polyembryony, with a specific allele associated with a MITE transposon insertion in the promoter of this gene[31] in the mandarin (*C. reticulata*) lineage. This polyembryonic allele was absent in other citrus species including pummelos and citrons. In *Poncirus trifoliata* however, apomixis appears to be controlled by a different molecular mechanism[62]. In this work, we will focus on apomixis in mandarins and interspecific mandarin hybrids.

To examine the haplotype diversity of the MITE transposon of the polyembryonic allele, we mapped Illumina short reads of sequenced mandarins and mandarin hybrids to the sweet orange reference sequence[6] which contains the MITE insertion. Four MITE haplotypes containing five segregating SNPs are identified (**Fig. 4a**, **Supplementary Fig. 8a, 8b**). Three of the five SNPs divide the 4 haplotypes into two haplogroups, H1 and H2. Within each haplogroup, one single SNP further differentiates between two haplotypes of each group. The occurrence of MITE alleles varies between groups and among types, with H1 group much more prevalent than H2. Specifically, MITE haplotype H2A is observed in only 6 Chinese mandarins (not counting clonal relatives) whereas type H2B is unique to yuzu (**Fig. 4a**). By contrast, the MITE insertion is absent in other citrus species including *C. ryukyuensis*, pummelos, citrons, as well as all monoembryonic mandarins including Clementine and kishu,

Interestingly, both MITE haplogroups H1 and H2 are present in the two wild mandarins from Mangshan (MS1, MS2). In particular, MS1 is heterozygous for the MITE insertion and carries H2A haplotype, whereas MS2 is the only known citrus containing both H1A and H2A haplotypes. The MITE haplotypes for the sequenced mandarins, oranges, grapefruit, lemons and other mandarin hybrids are listed in **Supplementary Data 1 & 3**, with representative accessions for each haplotype shown in **Fig. 4a**.

Apomixis originated from mangshanyeju

To investigate the genetic ancestry of the polyembryonic allele and its possible connection to the mangshanyeju population, the 200 kb region flanking the *CitRKD1* gene (Ciclev10010497m) (scaffold_1: 25380489-25582037 of the Clementine reference sequence[32]) was examined in a collection of 55 citrus accessions including mostly mandarins and interspecific mandarin hybrids and admixtures derived from six citrus species (PU=pummelo, CI=citron, IC=Ichang papeda, FO=*Fortunella*, RK=*C. ryukyuensis* and *C. reticulata*). Genetic admixture analysis was performed using ADMIXTURE[2] for K=3-11. For each K value, twenty independent runs were conducted and the run with lowest cross-validation error was used. The population ancestry

composition at K=8 is shown in **Fig. 4b**, with additional plots for K=8-10 included in **Supplementary Fig. 8c**.

With eight ancestral populations (K=8, **Fig. 4b**), all six citrus species are resolved with further differentiation of mandarins (*C. reticulata*) into 3 sub-populations, namely, common mandarin (MA), and two sub-populations of mangshanyeju h1 and h2. The identification of h1 and h2 with sub-populations of MS is based on the ancestry makeup of the two MS accessions MS1 and MS2. MS1 is the only citrus type with a pure h2 ancestry based on ADMIXTURE, whereas MS2 derives its genetic ancestry from both h1 and h2. Furthermore, a surprisingly simple correspondence can be established between h1 and MITE haplogroup H1 and between h2 and MITE haplogroup H2 respectively, after careful examination of the ancestry composition in **Fig 4b** and MITE allele of each accession. Thus, the population stratification within MS at this extended locus of 200kb is supported by the haplotype structure of the MITE alleles.

Insight can also be obtained on the origin and dispersal of the apomixis alleles based on the genetic ancestry makeup in **Fig. 4b**. Among mandarins and interspecific mandarin hybrids, all polyembryonic accessions have either h1 and/or h2 MS ancestry, whereas all monoembryonic accessions without MITE insertion in the promoter of *CitRKD1* derive their mandarin ancestry from common mandarin rather than the MS subspecies. The MITE insertion is absent in the five non-mandarin species of **Fig. 4b** including *C. ryukyuensis*. These observations provide strong evidence that the polyembryonic allele associated with the MITE insertion originated from the mangshanyeju subspecies, with subsequent introgression into common mandarins as well as interspecific hybrids and admixtures including oranges, grapefruit, lemons, Rangpur lime, rough lemon, yuzu, calamondin, as well as admixed accessions with *C. ryukyuensis* ancestry (namely tachibana, shiikuwasha, the yukunibu group, rokugatsu and deedee).

As a fine detail, the MITE insertion is not fixed in mangshanyeju (as MS1 is heterozygous for the MITE insertion), it is possible for a common mandarin or mandarin hybrid to have MS ancestry at the apomixis locus without carrying the polyembryonic MITE allele. Interestingly, this occurs only in one of the sequenced accessions, M04 (suanpangan wild mandarin), where the 200 kb locus has both h1 and h2 ancestry but contains only one copy of the MITE allele (H1B) since the MITE insertion is absent in the haplotype with h2 ancestry. Note also that three polyembryonic accessions including keraji-mikan have pure h1 MS ancestry at this locus (without common mandarin ancestry), they each contain two identical MITE H1 alleles due to parental haplotype sharing (backcross in the case of kerajji). The rarity of monoembryonic mandarins with MS admixture at the apomixis locus can be explained by the extreme selection pressure in favor of nucellar polyembryony due to its capacity for clonal expansion.

With higher numbers of ancestral populations, progressively finer resolution is observed for K=9 and 10 (**Supplementary Fig. 8c)**. At K=9, two sub-populations (m1, m2) are differentiated within common mandarins. With ten ancestral populations (K=10), further differentiation within pummelos (PU, p2) is revealed. Importantly, the two mangshanyeju (h1, h2) sub-population ancestry compositions remain unchanged for K=8-10.

An early Pleistocene origin for apomixis

It was previously observed that the MITE insertion associated with the dominant polyembryonic allele arose in the mandarin (*C. reticulata*) lineage.[31] With the discovery of the monoembryonic *C. ryukyuensis* species, the origin of apomixis is placed after the divergence between *C. ryukyuensis* and *C. reticulata* 2.2-2.8 Mya (**Fig. 2a**, **Supplementary Note 11**). The identification of the mangshanyeju ancestry of the apomixis alleles further constrains the rise of apomixis no earlier than the divergence between mangshanyeju and common mandarins 1.4-1.7 Mya (**Fig. 2a**, **Supplementary Note 11**). To obtain a lower bound on the timing of apomixis however, we need to examine the genetic structure of the apomixis locus.

The MITE haplotype structure (**Fig. 4a**) indicates that the MITE insertion arose only once in the mangshanyeju population after its divergence from common mandarins, with subsequent diversification due to somatic mutations giving rise to two haplogroups containing four haplotypes. The sequence divergence between the two MITE haplogroups is 2% (based on the average pairwise differences between H1 and H2 among the four haplotypes for the approximately 200bp[31,40] MITE sequence). This is comparable to citrus inter-specific divergence and suggests an ancient origin for apomixis. The sub-population differentiation within mangshanyeju at the extended 200kb locus (**Fig. 4b**) provides further support for the ancient origin model of apomixis, in which a lower bound on the timing of first MITE insertion is placed before the divergence of the two MITE haplogroups.

Although an accurate calculation of the lower bound on the MITE insertion time is not possible due to the lack of knowledge of base substitution rate in citrus and the small target size of the MITE, a rough estimate can be made using the silent base substitution rate in angiosperms ($5\text{-}7 \times 10^{-9}$ per site per year[63,64]). This translates into a time for the most recent common ancestor (TMRCA) of the two MITE haplogroups around 1.4-2.0 Mya, with 95% confidence interval of 0.4-4 Mya (assuming a Poisson process). This lower bound on the origin of apomixis overlaps significantly (with) the upper bound derived from the subspecies divergence between MS and MA 1.4-1.7 Mya. Taken together, they point to an early Pleistocene origin of apomixis, likely not long after the split between mangshanyeju and common mandarins.

Although we have only two diploid mangshanyeju accessions (MS1, MS2) plus three MS haploid genomes (from yuzu, M01, M04), these seven haploid MS genomes contain all four MITE haplotypes (in two haplogroups) (**Fig. 4a**). On one hand, it suggests that introgression of the MITE alleles into common mandarins and mandarin hybrids occurred much more recently than the origin of the apomixis allele, probably after the diversification of the MITE haplotypes within mangshanyeju. On the other hand, this points to a possibly much greater diversity at the apomixis locus yet to be explored. Additional sequencing of the MS diversity is necessary to gain a better understanding of the population structure of mangshanyeju, the full haplotype diversity of the MITE alleles, and the origin and evolution of apomixis.

Loss of anthocyanin biosynthesis in mandarin-type citrus

For mandarin-type citrus, anthocyanin production is absent in leaves and flowers due to nonfunctional alleles of the MYB transcription factor called *Ruby*[65,66], which are required for anthocyanin biosynthesis in citrus. Within our evolutionary framework, we can trace the origin of defective *Ruby* alleles. The *Ruby* gene (Ciclev10013455m, scaffold_6:22,492,532-22,494,209) contains three exons and is located at the end of chromosome 6 of the Clementine reference

sequence[32], where pummelo admixture is observed in many cultivated mandarins[3]. The *Ruby* allele in the haploid Clementine reference sequence has pummelo ancestry based on admixture pattern[32]. This allele is defective due to a premature stop mutation (r^{STOP}) in the third exon[65]. This r^{STOP} allele is also found in Ponkan, Willowleaf and some other mandarins with pummelo admixture at the *Ruby* locus, and likely originated from an early phase of introgression involving a single pummelo tree as hypothesized for type 2 mandarins[3]. As noted previously[65,67], functional *Ruby* alleles with pummelo ancestry are extremely rare, and only defective *Ruby* alleles of pummelo ancestry have been observed in cultivated pummelos and admixed mandarins.

Ruby alleles with mandarin (*C. reticulata*) ancestry have been examined in several cultivated mandarins[65]. Two overlapping deletion alleles have been described ($r^{DEL0.8}$ and r^{DEL2}, with deletion sizes of 0.8kb and 2kb respectively) and found defective due to the deletion of the first two exons in both alleles. The r^{DEL2} allele may have arisen as a mutation of $r^{DEL0.8}$, since its deletion sequence encompasses $r^{DEL0.8}$. Interestingly, only $r^{DEL0.8}$ is observed in the wild mandarins of mangshanyeju. Furthermore, the same allele ($r^{DEL0.8}$) is found fixed in *C. ryukyuensis*, indicating that its origin might predate the divergence of *C. ryukyuensis* and mainland Asian mandarins 2.2-2.8 Mya. By contrast, both deletion alleles are observed in the common mandarin population, suggesting a later origin for the larger, overlapping deletion as a derived allele of $r^{DEL0.8}$.

Current sequencing data support that all *Ruby* alleles with mandarin-type ancestry have lost their first two exons and are thus defective. The ancient origin of the deletion allele $r^{DEL0.8}$ explains the widespread loss of anthocyanin biosynthesis in both mainland Asian and Japanese mandarins. Our analysis indicates that the loss of *Ruby* activity in mandarin-type citrus is not a result of, but rather predates, domestication and human selection[67]. As *Ruby* controls the pigmentation of petals[68], these ancient nonfunctional alleles are the genetic cause for the appealing white flowers of oranges, tachibana and shiikuwasha as reflected in ancient poetry.

Supplementary Note 11. Timings of population divergence

Divergence of *C. ryukyuensis*, mangshanyeju and common mandarins

The genetic diversity of east Asian citrus, and Ryukyuan citrus in particular, derives from four major strongly differentiated populations, PU=pummelos (*C. maxima*), RK=*C. ryukyuensis*, and two mandarin (*C. reticulata*) populations, namely, MS=mangshanyeju (Mangshan wild mandarins) and what we refer to as common mandarins (MA). Based on admixture analysis of sequenced genomes, pummelos and *C. ryukyuensis* populations show no or little interspecific admixture. Similarly, the wild MS population, located in Southern Hunan province of China and represented by two recently sequenced accessions, shows no sign of admixture. By contrast, common mandarins are a heterogenous group with widespread admixture from both pummelos and mangshanyeju (**Fig. 1b**, **Supplementary Note 9**).

To estimate the divergence times and effective population sizes of these four populations, we selected two accessions from each population and excluded admixed regions of the genome. Specifically, Sun Chu Sha Kat (SCM) and Cleopatra (CLP) were used to represent MA. The folded joint allele frequency spectrum (AFS) of the 4 populations was derived from the eight chosen samples and fitted with population genetic models of demography. Demographic inference was conducted using moments[4], a python package that can efficiently simulate multidimensional AFS and infer demographic history. Due to both the high dimensionality of the joint AFS and small sample sizes, we restricted to ten-parameter 4-population models with piecewise constant population sizes but no between-population migrations (**Supplementary Fig. 5**). Based on the robust topology of the reconstructed phylogenetic tree (**Supplementary Fig. 2a**), the demographic model with the specific topology (PU, (RK, (MA, MS))) is used to describe the speciation process.

For time calibration, the PU/MA divergence was constrained to be in the range 6-7.5 Mya based on our previous analysis[3] and the citrus leaf fossil record from the late Miocene[69]. The timing is also in agreement with earlier molecular dating of the orange subfamily Aurantioideae using cpDNA[70] and a few nuclear loci[71], with non-citrus fossil calibrations. For effective population size estimates, we used a generation time of 10 years. For moments simulations, multiple runs were performed with independent starting points in the ten-dimensional parameter space and checked for convergence of the likelihood and model parameter values. The estimated model parameters are listed in **Supplementary Fig. 5b** with uncertainties reflecting the time calibration range of the PU/MA divergence.

The initial phase of Asian citrus radiation occurred during the late Miocene 6-8 Mya with subsequent migration to Oceania in the early Pliocene circa 4 Mya[3]. We find that later speciation events during the early Pleistocene gave rise to *C. ryukyuensis* 2.2-2.8 Mya and the divergence of the two subspecies of *C. reticulata* (MS and MA) 1.4-1.7 Mya. The early Pleistocene origin of *C. ryukyuensis* is consistent with the geological history of the Ryukyu Arc (**Fig. 2b**), which had been part of the Asia continent during the late Miocene (11.6-5.3 Mya) and part of the Pliocene (5.33-2.58 Mya). Later tectonic activity and sea level changes caused the formation of the Ryukyu Arc and the East China Sea[72,73]. The variable connectivity of the Ryukyu Arc to the

Asian mainland associated with climatic variations during the Pleistocene led to the emergence of *C. ryukyuensis* and other new species in the region[74-77].

The observed admixture pattern in common mandarins (**Fig. 1b**) indicates later gene flow from both pummelos and Mangshan wild mandarins. The genomics-informed genealogy of extant citrus varieties in the Ryukyu Islands and mainland Japan (**Fig. 3a; Supplementary Fig. 7**) further indicates that some of the admixed mainland Asian mandarins arrived to the Ryukyu Arc and mainland Japan during different time periods. Subsequent hybridizations of these migrant mandarin populations with *C. ryukyuensis* gave rise to the diverse citrus types of tachibana in mainland Japan, and shiikuwasha, the yukunibu group and other indigenous varieties in the Ryukyu Islands. Although *C. ryukyuensis* is monoembryonic, these hybrid types are apomictic by inheriting the alleles for nucellar polyembryony from their mainland Asian mandarin ancestors.

A population bottleneck for *C. ryukyuensis*

The effective population size of *C. ryukyuensis* is estimated at 56,000-70,000, which is about 1/3 of the size of the common ancestor of *C. ryukyuensis* and *C. reticulata* (156,000-195,000) and half of the size of mangshanyeju (104,000-130,000). The reduced population size of *C. ryukyuensis* is consistent with its lower nucleotide diversity ($0.2\text{-}0.3\times10^{-3}$), about half of the observed nucleotide diversity in mangshanyeju or common mandarins. When combined with the deep divergence times, this implies a severe population bottleneck for *C. ryukyuensis*, the intensity of which can be measured by the inbreeding coefficient[78]

$$F = 1 - e^{-\frac{T_{RK}}{2N_{RK}}} = 1 - e^{-2.0} = 0.86 \qquad (1)$$

where T_{RK} is the divergence time of *C. ryukyuensis* in generations and N_{RK} is its effective population size. This population bottleneck could be due to the founder effect when the *C. ryukyuensis* population was isolated from mainland Asian mandarins during most of the Pleistocene epoch, with resultant allopatric speciation.

By contrast, the effective population size of the common ancestor of pummelos and mandarins during late Miocene is estimated at 482,000-602,000, which is about nine times the size of *C. ryukyuensis* and about five times of the size of mangshanyeju. This large ancestral population size indicates a much greater genetic diversity near the origin of citrus 6-7.5 Mya and is in line with the late Miocene citrus radiation giving rise to at least seven species in a time span of 2 million years[3].

Divergence of two *C. ryukyuensis* sub-populations

The geographical separation of mainland Japan tachibana from Okinawa indigenous citrus types suggests the possibility of differentiation between the Okinawan *C. ryukyuensis* population and the *C. ryukyuensis* ancestors of tachibana. To estimate Fst between these two *C. ryukyuensis* sub-populations, we represent the mainland Japan population by phased *C. ryukyuensis* haplotypes derived from inter-specific hybrid regions of the tachibana genomes. The weighted

Fst based on vcftools[34] between Okinawa and tachibana-derived *C. ryukyuensis* populations is estimated to be 0.20.

As a complementary approach, the mainland Japan *C. ryukyuensis* population can also be approximated by the diploid *C. ryukyuensis* segments of tachibana. For this purpose, we used the tachibana accession TB5, which has the highest proportion (18% or 54 Mb) of diploid *C. ryukyuensis* among the three genomes. The resultant Fst between mainland Japan and Okinawa *C. ryukyuensis* sub-populations is 0.17, comparable to the estimate of 0.20 based on hybrid genomic regions of tachibana.

To estimate the divergence time between the two *C. ryukyuensis* sub-populations, we conducted coalescent simulations using macs[79] to match the observed Fst = 0.17-0.20. In the simplest 2-parameter isolation model with two populations descending from an ancestral population T generations ago, we found that T/(2N) = 0.20-0.25 where N is the effective population size for both the extant and ancestral populations. Using the effective population size of the extant *C. ryukyuensis* population N=56,000-70,000 and generation time of 10 years, the estimated divergence time between the Okinawan and tachibana-progenitor *C. ryukyuensis* populations is T=220-350 kya.

For an alternative estimate of the divergence time between mainland Japan and Ryukyuan *C. ryukyuensis* populations, we added the tachibana-derived *C. ryukyuensis* population to the 4-population model **introduced at the beginning of this section** and used moments[4] to simulate the joint AFS of the five populations. As moments requires at least two diploid samples for each population, we used the genomic regions where both TB5 and M00 are diploid *C. ryukyuensis* to represent the mainland Japan tachibana-progenitor population. After excluding admixture regions in all ten samples used to represent the five populations, the sequence space is reduced to 3.9 Mb or about 1.3% of the genome. By fixing the demographic parameters of the 4-population model as inferred **earlier in this section** and assuming the same effective population sizes for the two *C. ryukyuensis* populations, the divergence time is estimated to be 190-240 kya. As this inference is based on a small fraction of the genome, it may not represent an unbiased estimate of the divergence time. Nevertheless, the estimated time span is consistent with the above Fst-based estimate.

We also noted a reduction in heterozygosity in the tachibana-progenitor *C. ryukyuensis* relative to the Okinawa population. More specifically, the heterozygosity of mainland Japan population is estimated using the diploid *C. ryukyuensis* segments in each of three tachibana genomes and compared to the mean heterozygosity of the eight Okinawa accessions for the same genomic region. The ratio of heterozygosity is estimated at 0.87, 0.93, and 0.85 based on the three tachibana accessions of TB0, TB5 and M00 respectively. Thus, the heterozygosity of the *C. ryukyuensis* population ancestral to tachibana is 7-15% lower than the Okinawa population, indicative of a modest bottleneck.

We find no significant genetic differentiation between the Okinawa *C. ryukyuensis* population and the *C. ryukyuensis* haplotypes derived from shiikuwasha or the yukunibu group. This is not unexpected given their shared habitat and is in line with the recent introduction of kunenbo to the

Ryukyu Islands. This also suggests a putatively recent but still pre-historic origin of shiikuwasha, although we cannot make a precise estimate of this timing.

In summary, there is significant genetic differentiation between the Okinawa *C. ryukyuensis* population and the presumptive *C. ryukyuensis* population that gave rise to tachibana in mainland Japan. Furthermore, the 7-15% reduction in heterozygosity for the mainland Japan *C. ryukyuensis* population relative to the Okinawa population is consistent with a presumed south-to-north dispersal of *C. ryukyuensis* and the founder effect.

The origin of tachibana likely predates shiikuwasha

The population structure of *C. ryukyuensis* can inform us about the pre-historical origins of the tachibana and shiikuwasha. The genetic homogeneity among the *C. ryukyuensis* ancestors of shiikuwasha and the yukunibu group, together with the historical record on the introduction of kunenbo (maternal parent of the yukunibu group) to the Ryukyu Islands, indicates that the origin of shiikuwasha may be no more than a few thousand years old. This is in line with the fact that a clonal relative of the direct parent of shiikuwasha, a type 2 mainland Asian mandarin with small amount of pummelo admixture[3], can still be found in Okinawa.

By comparison, the significant genetic differentiation between tachibana-derived *C. ryukyuensis* haplotypes and the Okinawa *C. ryukyuensis* population points to an independent and ancient origin for mainland Japan tachibana. These two differences between tachibana and shiikuwasha together suggest a more ancient origin for tachibana than shiikuwasha. (1) The genomic admixture pattern of tachibana (**Fig. 3b**) required a more complex hybridization process than the simple F1 crosses at the origin of shiikuwasha. (2) The mainland Asian mandarin ancestor of tachibana is inferred to be a mandarin hybrid with comparable ancestry from mangshanyeju and common mandarin, but without pummelo admixture. This suggests that tachibana originated after the divergence of the mainland Japan and Okinawa populations of *C. ryukyuensis* populations 220,000-350,000 years ago but plausibly before the widespread pummelo introgression in mainland Asian mandarins that is associated with domestication[3,80] By contrast, the observed pummelo segment in RK3 necessitates a post pummelo-introgression origin for shiikuwasha.

Supplementary Note 12. An admixture-informed classification of mandarin-type citrus

An evolutionary framework for classifying mandarin diversity

The discovery of *C. ryukyuensis* illuminates the genetic ancestries of tachibana, shiikuwasha and other varieties indigenous to the Ryukyu Islands. The evolutionary history of east Asian mandarin-type citrus reveals a complex admixture process involving four populations, with both mangshanyeju and pummelo introgression among common mandarins, followed by distinct hybridizations of *C. ryukyuensis* with a few mainland mandarins, the sour orange and pummelo. The resulting complex admixture patterns, combined with the apomixis trait, have contributed to the extreme heterogeneity of mandarin-type citrus, and had caused great confusion in the classification of mandarins prior to the genomics era. On the other hand, genomics-informed admixture patterns in mandarins provide an evolutionary framework to classify mandarin-type citrus, as has been proposed previously[3] based primarily on the size of pummelo ancestry in mandarins. Under this classification scheme, mainland Asian mandarins as well as tachibana were grouped into three types corresponding to no, little (a few percent), and significant pummelo admixture. In particular, both tachibana and some Chinese wild mandarins without pummelo admixture were placed under type 1.

To take into account the differentiation among mangshanyeju, common mandarin and *C. ryukyuensis*, we update our previously proposed classification[3] by introducing a new 'type 4' mandarin notation to describe varieties with *C. ryukyuensis* ancestry and use subtypes to designate different hybrid/admixture groups. Mangshanyeju represents a new subtype of pure mandarins (*C. reticulata*). Some representative accessions are listed in parenthesis. Note that this classification of mandarin-type citrus is distinct from Yamamoto's classification of citrus cpDNA[15].

Type 1a or mangshanyeju mandarin refers to wild mandarins (*C. reticulata*) from Mangshan[49] without pummelo admixture.

Type 1b mandarin applies to other mandarins without pummelo admixture, including hybrid MS/MA wild mandarins (M01, M04) and common mandarins with MS admixture (M02, SCM).

Type 2 or early admixture mandarins are common mandarins with small amount of pummelo ancestry (usually a few percent), likely derived from an ancient introgression involving a single pummelo[3]. This group includes both acidic (CLP, SNK, RK3, M03) and palatable varieties (PKM, DNC, KSH).

Type 3 or late admixture mandarins refer to common mandarins with a significant amount of pummelo admixture due to additional pummelo introgression. This group includes most if not all of the modern mandarins as well as other older palatable varieties (king, satsuma, kunenbo). Mandarin hybrids with sweet orange (tangors) and grapefruit (most tangelos) also belong to this group.

The new Type 4 comprises of mandarins with *C. ryukyuensis* ancestry, with subtypes corresponding to different hybrid groups as follows:

Type 4a or *C. ryukyuensis* described here refers to different tanibuta accessions from Okinawa with minimal interspecific admixture.

Type 4b or tachibana contains tachibana from mainland Japan with ancestry derived from *C. ryukyuensis* and a presumptive type 1b Chinese wild mandarin, itself a hybrid of mangshanyeju and common mandarin.

Type 4c or shiikuwasha resulted from F1 hybridizations between *C. ryukyuensis* and the type 2 Chinese mandarin RK3. Six half-sib varieties from Okinawa were identified in our collection.

Type 4d or yukunibu group refers to the extended half-sib family derived from hybridizations of kunenbo (*C. nobilis* Lour. var. *kunip* Tanaka, a type 3 mandarin) and *C. ryukyuensis* and derivatives thereof. Five half-sib accessions from Okinawa are in our collection including kabuchii, oto, tarogaro. Also included in this group are two kabuchii derivatives, keraji (kunenbo × kabuchii) and a kaneshi variety (R07=kabuchii × shiikuwasha).

Type 4e or rokugatsu refers to hybrid varieties of *C. ryukyuensis* × sour orange. Our collection contains one accession from Okinawa (RKG=zadaidai).

The Ryukyu sour orange, Nakijin-deedee (R00), is a hybrid of pummelo × shiikuwasha. Like the common sour orange (pummelo × mandarin F1 hybrid), we do not attempt to fit Ryukyu sour orange into a mandarin-type citrus classification scheme.

The mandarin divergence and the consequences for citrus domestication.

The divergence between *C. reticulata* and *C. ryukyuensis* in east Asia occurred 2.2-2.8 Mya, which overlaps the Pliocene-Pleistocene boundary 2.6 Mya. Mangshanyeju and common mandarin diverged during the Pleiostocene 1.4-1.7 million years ago (**Fig. 2a**). The previous epoch, the Pliocene, was a generally warmer and wetter period that gave way to early Pleistocene cooling. Throughout this geological epoch, large continental ice sheets in the Northern Hemisphere became widespread leading to the establishment of cool conditions that might well have contributed to new citrus speciation, as suggested for the Late Miocene citrus radiation[3].

It is generally accepted that major changes in the flora and fauna during the Pliocene and Pleistocene epochs were mediated by changes or shifts in climate. African climate variability, for instance, reached maxima at 2.8 and 1.7 Mya coincident with the intensification of high-latitude glacial cycles and with higher mammal fossil registers that were also dated at 2.4-2.9 Mya and 1.6-1.8 Mya[81]. It has been also suggested[82] that the climate became colder and exhibited greater temperature variations throughout the Pleistocene. In coastal Asia, sea levels rose and fell according to the succession of repeated glaciations, opening during the interglacial phases major opportunities for allopatric speciation of *C. ryukyuensis* and migration of mainland Asian mandarins to the Taiwan and Ryukyu islands (**Fig. 2b**). There is evidence, for instance, that during the Late Miocene continental Asia also extended to include the Ryukyu region, and after the warm Pliocene, during the Pleistocene, this arc was several times connected to mainland Asia

through Taiwan[72,73] that acted as a land bridge for dispersion of mainland flora and fauna. It is also worth noting that it is now commonly accepted that the evolution of biodiversity on nearby islands is consistently linked to transoceanic seed dispersal that can effectively reach long distances via ocean currents[83]. Dispersal is fundamental to biogeography and the evolution of biodiversity on oceanic islands. Therefore, the dispersal of these ancestors of mainland mandarins through Taiwan, the Ryukyu Arc and mainland Japan could also have been achieved by transoceanic seeds carried by the strong Kuroshio Current[77,84] that crosses these regions from south to north before turning towards the open Pacific Ocean.

Remarkably, climate changes during the Pleistocene had major consequences not only on citrus evolution but deeply shaped the biology of domesticated citrus. It has been reported that the late Miocene citrus radiation[3], a period of major climatic alterations, was accompanied by a strong reprograming of transposon activity, a mechanism proposed to respond to stressful conditions driving speciation as a part of the adaptive response[85]. Similarly, the MITE transposon insertion described above also occurred in a phase of the Pleistocene that exhibited greater temperature variations and was roughly coincident with the mangshanyeju and common mandarin split. This insertion is associated with apomixis, a trait that tends to conserve the maternal genetic structure by rapidly fixing its genotype and conferring great advantage for its widespread diffusion.

As progress in the elucidation of the citrus genome is advancing it is becoming evident that citrus domestication occurred through two independent ancestral introgressions: the gain of apomixis[31,40] provided by mangshanyeju, and the pummelo introgression that incorporated desirable traits into the ancestral mandarin genome[32]. Genomic evidence also supports that after these pivotal domestication events there were numerous crosses involving those ancestral hybrids and admixtures, giving rise to the current basic types of edible citrus[3,86]. Later, clonal propagation presumably accelerated the multiplication and diffusion of superior individuals bearing better organoleptic characteristics and valuable agronomic traits.

Supplementary references

1 Stamatakis, A. RAxML version 8: a tool for phylogenetic analysis and post-analysis of large phylogenies. *Bioinformatics* **30**, 1312-1313, doi:10.1093/bioinformatics/btu033 (2014).

2 Alexander, D. H., Novembre, J. & Lange, K. Fast model-based estimation of ancestry in unrelated individuals. *Genome Res* **19**, 1655-1664, doi:10.1101/gr.094052.109 (2009).

3 Wu, G. A. *et al.* Genomics of the origin and evolution of *Citrus*. *Nature* **554**, 311 (2018).

4 Jouganous, J., Long, W., Ragsdale, A. P. & Gravel, S. Inferring the joint demographic history of multiple populations: beyond the diffusion approximation. *Genetics* **206**, 1549-1567, doi:10.1534/genetics.117.200493 (2017).

5 Robinson, J. T. *et al.* Integrative genomics viewer. *Nat Biotechnol* **29**, 24-26, doi:10.1038/nbt.1754 (2011).

6 Xu, Q. *et al.* The draft genome of sweet orange (*Citrus sinensis*). *Nat Genet* **45**, 59-66, doi:10.1038/ng.2472 (2013).

7 Yamamoto, M., Takakura, A., Tanabe, A., Teramoto, S. & Kita, M. Diversity of *Citrus depressa* Hayata (Shiikuwasha) revealed by DNA analysis. *Genetic Resources and Crop Evolution* **64**, 805-814 (2017).

8 Iwamasa, M., Ueno, I. & Nishiura, M. Inheritance of nucellar embryony in citrus. *Bull. Hort. Res. Stn.* **7**, 1-10 (1967).

9 Kobayashi, S., Ikeda, I. & Nakatani, M. Studies on nucellar embryogenesis in citrus. *Journal of the Japanese Society for Horticultural Science* **48**, 179-185 (1979).

10 Wakana, A. & Uemoto, S. Adventive embryogenesis in citrus I. The occurrence of adventive embryos without pollination or fertilization. *American Journal of Botany* **74**, 517-530 (1987).

11 Tanaka, T. Fundamental discussion of *Citrus* classification. *Studia Citrologica* **14**, 1-6 (1977).

12 Tanaka, T. A revision of Ryukyu *Rutaceae-Aurantioideae*, Revisio Aurantiacearum X. *Sci bull Agr Home Eco Division, University of the Ryukyus* **4**, 91-116 (1957).

13 Swingle, W. T. & Reece, P. C. in *The citrus industry, revised 2nd ed., History, world distribution, botany, and varieties* Vol. 1 (eds W. Reuther, H.J. Webber, & L.D. Batchelor) Ch. 3, 190–430 (University of California, Berkeley, California, USA, 1967).

14 Yamamoto, M., Matsuo, Y., Kuniga, T., Matsumoto, R. & Yamada, Y. Isozyme and RAPD analyses of Shiikuwashas (*Citrus depressa* Hayata). *Bulletin of the Fruit Tree Research Station (Japan)* (1998).

15 Yamamoto, M. *et al.* Diversity of chloroplast DNA in various mandarins (*Citrus spp.*) and other citrus demonstrated by CAPS analysis. *Journal of the Japanese Society for Horticultural Science* **82**, 106-113 (2013).

16 Hirai, M., Mitsue, S., Kita, K. & Kajiura, I. A survey and isozyme analysis of wild mandarin, tachibana (*Citrus tachibana* (Mak.) Tanaka) growing in Japan. *Journal of the Japanese Society for Horticultural Science* **59**, 1-7 (1990).

17 Inafuku-Teramoto, S. *et al.* Local citrus genetic resources and their polymethoxyflavones content in northern part of Okinawa Island. *Horticultural Research (Japan)* **9**, 263-271 (2010).

18 Deng, X., Yang, X., Yamamoto, M. & Biswas, M. K. in *The Genus Citrus* (eds M. Talon, M. Caruso, & F.G. Gmitter) 33-55 (Elsevier, 2020).

19 Shimizu, T. *et al.* Hybrid origins of citrus varieties inferred from DNA marker analysis of nuclear and organelle genomes. *PLoS One* **11**, e0166969, doi:10.1371/journal.pone.0166969 (2016).
20 Tanaka, T. Citrus fruits of Japan: with notes on their history and the origin of varieties through bud variation. *Journal of Heredity* **13**, 243-253 (1922).
21 Omodaka, H. *Man'yōshū with Annotations* p 115 (in Japanese) (Dai Nihon Insatsu, Tokyo, Japan, 1958).
22 Sullivan, S. L., deWolf, G. P. J., Potter, G. E. & Brooks, E. B. Plants in early Japanese poetry. *Arnoldia* **31**, 283-293 (1971).
23 Tanaka, Y. *An iconography of Japanese citrus fruits [in Japanese]*. Vol. 2 (Yokendo, Tokyo 1948).
24 Onishi, M. & Miyagi, K. *Wisdom in Shiikuwasha-Oku/ Yanbaru, Circulation among dialect, local community, life.*, 529 (Kyoto University Press, 2016).
25 Ishikawa, R. *et al.* Multi-lineages of Shiikuwasha (*Citrus depressa* Hayata) evaluated by using whole chloroplast genome sequences and its bio-diversity in Okinawa, Japan. *Breeding Science*, 15151 (2016).
26 Grosser, J., Gmitter, F. & Bowman, K. New rootstocks in the citrus breeding pipeline. *Citrus Industry* **101**, 8-11 (2020).
27 Hokama, S., Hateruma, E. . *The Standard Text of Omoro Sōshi* p 13-16, p 18 (Kadokawashoten, Tokyo, Japan (in Japanese) 2002).
28 Nakahara, Z. & Hokama, S. *Omoro Sōshi Dictionary and Index*. 127 (1967).
29 Shimizu, A. *Omoro Sōshi with Annotations and Transcriptions of Speech Sound Vol. 1*. 29pp, 246pp (Izumi Shoin, Osaka, Japan (in Japanese), 2003).
30 Iha, F. *Omoro soshi senshaku (Anthology and Annotations of Omoro soshi) p112* (Ishizuka Shoten, Okinawa, Japan (in Japanese), 1924).
31 Wang, X. *et al.* Genomic analyses of primitive, wild and cultivated citrus provide insights into asexual reproduction. *Nature Genetics* **49**, 765 (2017).
32 Wu, G. A. *et al.* Sequencing of diverse mandarin, pummelo and orange genomes reveals complex history of admixture during citrus domestication. *Nat Biotechnol* **32**, 656-662, doi:10.1038/nbt.2906 (2014).
33 Weir, B. S. & Cockerham, C. C. Estimating F-statistics for the analysis of population structure. *Evolution* **38**, 1358-1370, doi:10.1111/j.1558-5646.1984.tb05657.x (1984).
34 Danecek, P. *et al.* The variant call format and VCFtools. *Bioinformatics* **27**, 2156-2158, doi:10.1093/bioinformatics/btr330 (2011).
35 Tanaka, Y. *An iconography of Japanese citrus fruits*. Vol. 2 (1948).
36 Medoruma, K. *et al.* Characteristics of seedless *Citrus depressa*, Nakamoto seedless. *Bull. Okinawa Pref. Agric. Res. Center* (2011).
37 Stern, C. Somatic crossing over and segregation in *Drosophila melanogaster*. *Genetics* **21**, 625 (1936).
38 LaFave, M. C. & Sekelsky, J. Mitotic recombination: why? when? how? where? *PLoS Genetics* **5** (2009).
39 Chen, L. Y. *et al.* The bracteatus pineapple genome and domestication of clonally propagated crops. *Nat Genet* **51**, 1549-1558, doi:10.1038/s41588-019-0506-8 (2019).
40 Shimada, T. *et al.* MITE insertion-dependent expression of CitRKD1 with a RWP-RK domain regulates somatic embryogenesis in citrus nucellar tissues. *BMC Plant Biol* **18**, 166, doi:10.1186/s12870-018-1369-3 (2018).

41 Garcia-Lor, A., Luro, F., Ollitrault, P. & Navarro, L. Genetic diversity and population structure analysis of mandarin germplasm by nuclear, chloroplastic and mitochondrial markers. *Tree Genetics & Genomes* **11**, 1-15 (2015).
42 Tanaka, T. The discovery of *Citrus tachibana* in Formosa, and its scientific and industrial significance. *Studia Citrologia* **5**, 1-20 (1931).
43 Tanaka, T. Citologia: semi-centennial commemoration papers on citrus studies. *Osaka: Citrologia Supporting Foundation* **114** (1961).
44 Yamamoto, M., Nesumi, H., Matsumoto, R. & Tominaga, S. Confusion between two mandarin (*Citrus spp.*) cultivars (Keraji and Kabuchi) that originated on the Amami Archipelago (in Japanese). *Bulletin of the Faculty of Agriculture, Kagoshima University* **53**, 15-19 (2003).
45 Zhou, X.-H. *et al.* Distribution and evolution of citrus with S 4 and/or S 5 gene alleles for self-incompatibility with special focus on the origin of satsuma mandarin (*Citrus unshiu* Marc.; S f S 4). *Genetic Resources and Crop Evolution* **65**, 1013-1033 (2018).
46 Ollitrault, P., Curk, F. & Krueger, R. in *The Genus Citrus* (eds M. Talon, M. Caruso, & F.G. Gmitter) 57-81 (Elsevier, 2020).
47 Yamamoto, M. Citrus genetic resources grown on the Ryukyu Islands, Japan. *南太平洋海域調査研究報告= Occasional papers*, 9-15 (2014).
48 Fujii, H. *et al.* Parental diagnosis of satsuma mandarin (*Citrus unshiu* Marc.) revealed by nuclear and cytoplasmic markers. *Breed Sci* **66**, 683-691, doi:10.1270/jsbbs.16060 (2016).
49 Wang, L. *et al.* Genome of wild mandarin and domestication history of mandarin. *Molecular Plant* **11**, 1024-1037 (2018).
50 Shimizu, T. *et al.* A model for the domestication and diversification processes of modern citrus cultivars in Japan. *Acta Hortic.* **1230**, 7-14 (2019).
51 Kajiura, I. *A chronological table of fruit history in Japan [in Japanese].* (Yokendo, Yokyo, 2008).
52 Tanaka, Y. *Developmental history of citrus varieties (1). Kankitsu.* 8: 308–315 (1941).
53 Tanaka, Y. *Developmental history of citrus varieties (2). Kankitsu.* 9: 5–16 (1942).
54 Abkenar, A. & Isshiki, S. Molecular characterization and genetic diversity among Japanese acid citrus (*Citrus spp.*) based on RAPD markers. *The Journal of Horticultural Science and Biotechnology* **78**, 108-112 (2003).
55 Matsumoto, T., Kimura, T. & Hayashi, T. Aromatic effects of a Japanese citrus fruit—yuzu *(Citrus junos* Sieb. ex Tanaka)—on psychoemotional states and autonomic nervous system activity during the menstrual cycle: a single-blind randomized controlled crossover study. *BioPsychoSocial Medicine* **10**, 11 (2016).
56 Karp, D. & Hu, X. The citron (*Citrus medica L.*) in China. *Horticultural Reviews* **45**, 143-196 (2018).
57 Ramadugu, C. *et al.* Genetic analysis of citron (*Citrus medica L.*) using simple sequence repeats and single nucleotide polymorphisms. *Scientia Horticulturae* **195**, 124-137 (2015).
58 Yang, X. *et al.* Genetic diversity and phylogenetic relationships of citron (*Citrus medica L.*) and its relatives in southwest China. *Tree Genetics & Genomes* **11**, 1-13 (2015).
59 Curk, F. *et al.* Phylogenetic origin of limes and lemons revealed by cytoplasmic and nuclear markers. *Annals of Botany* **117**, 565-583 (2016).

60 Lee, W. C. Testing the genetic relation between two individuals using a panel of frequency-unknown single nucleotide polymorphisms. *Annals of Human Genetics* **67**, 618-619, doi:10.1046/j.1529-8817.2003.00063.x (2003).
61 Nakano, M. *et al.* Characterization of genomic sequence showing strong association with polyembryony among diverse *Citrus* species and cultivars, and its synteny with *Vitis* and *Populus*. *Plant Science* **183**, 131-142 (2012).
62 Kepiro, J. & Roose, M. AFLP markers closely linked to a major gene essential for nucellar embryony (apomixis) in *Citrus maxima× Poncirus trifoliata*. *Tree Genetics & Genomes* **6**, 1-11 (2010).
63 Ossowski, S. *et al.* The rate and molecular spectrum of spontaneous mutations in *Arabidopsis thaliana*. *Science* **327**, 92-94, doi:10.1126/science.1180677 (2010).
64 De La Torre, A. R., Li, Z., Van de Peer, Y. & Ingvarsson, P. K. Contrasting rates of molecular evolution and patterns of selection among gymnosperms and flowering plants. *Molecular Biology and Evolution* **34**, 1363-1377 (2017).
65 Butelli, E. *et al.* Changes in anthocyanin production during domestication of *Citrus*. *Plant Physiol* **173**, 2225-2242, doi:10.1104/pp.16.01701 (2017).
66 Butelli, E. *et al.* Retrotransposons control fruit-specific, cold-dependent accumulation of anthocyanins in blood oranges. *Plant Cell* **24**, 1242-1255, doi:10.1105/tpc.111.095232 (2012).
67 Huang, D. *et al.* Subfunctionalization of the *Ruby2-Ruby1* gene cluster during the domestication of citrus. *Nat Plants* **4**, 930-941, doi:10.1038/s41477-018-0287-6 (2018).
68 Catalano, C. *et al.* Target-genes reveal species and genotypic specificity of anthocyanin pigmentation in *Citrus* and related genera. *Genes* **11**, 807 (2020).
69 Xie, S. P., Manchester, S. R., Liu, K. N., Wang, Y. F. & Sun, B. N. *Citrus Linczangensis Sp N.,* a leaf fossil of *Rutaceae* from the Late Miocene of Yunnan, China. *International Journal of Plant Sciences* **174**, 1201-1207, doi:10.1086/671796 (2013).
70 Pfeil, B. E. & Crisp, M. D. The age and biogeography of *Citrus* and the orange subfamily (*Rutaceae: Aurantioideae*) in Australasia and New Caledonia. *American Journal of Botany* **95**, 1621-1631, doi:10.3732/ajb.0800214 (2008).
71 Schwartz, T., Nylinder, S., Ramadugu, C., Antonelli, A. & Pfeil, B. E. The origin of oranges: a multi-locus phylogeny of *Rutaceae* subfamily *Aurantioideae*. *Systematic Botany* **40**, 1053-1062 (2016).
72 Kimura, M. Paleogeography of the Ryukyu Islands. *Tropics* **10**, 5-24 (2000).
73 Kimura, M. *The formation of the Ryukyu arc and migration of biota to the arc [in Japanese]*. (Okinawa Times, Naha, 2002).
74 Nakamura, K. *et al.* Phylogeny and biogeography of the *Viola iwagawae-tashiroi* species complex (*Violaceae,* section *Plagiostigma*) endemic to the Ryukyu Archipelago, Japan. *Plant Systematics and Evolution* **301**, 337-351 (2015).
75 Chiang, T.-Y. & Schaal, B. A. Phylogeography of plants in Taiwan and the Ryukyu Archipelago. *Taxon* **55**, 31-41 (2006).
76 Hiramatsu, M., Ii, K., Okubo, H., Huang, K. L. & Huang, C. W. Biogeography and origin of *Lilium longiflorum* and *L. formosanum* (*Liliaceae*) endemic to the Ryukyu Archipelago and Taiwan as determined by allozyme diversity. *American Journal of Botany* **88**, 1230-1239 (2001).
77 Nakanishi, H. Dispersal ecology of the maritime plants in the Ryukyu Islands, Japan. *Ecological Research* **3**, 163-173 (1988).

78 Blum, M. G. & Jakobsson, M. Deep divergences of human gene trees and models of human origins. *Mol Biol Evol* **28**, 889-898, doi:10.1093/molbev/msq265 (2011).
79 Chen, G. K., Marjoram, P. & Wall, J. D. Fast and flexible simulation of DNA sequence data. *Genome Research* **19**, 136-142, doi:10.1101/gr.083634.108 (2009).
80 Oueslati, A. *et al.* Genotyping by sequencing reveals the interspecific *C. maxima / C. reticulat*a admixture along the genomes of modern citrus varieties of mandarins, tangors, tangelos, orangelos and grapefruits. *PLoS One* **12**, e0185618, doi:10.1371/journal.pone.0185618 (2017).
81 DeMenocal, P. B. African climate change and faunal evolution during the Pliocene-Pleistocene. *Earth Planet Sc Lett* **220**, 3-24, doi:10.1016/S0012-821x(04)00003-2 (2004).
82 Emiliani, C. Pleistocene temperatures. *The Journal of Geology* **63**, 538-578 (1955).
83 Cowie, R. H. & Holland, B. S. Dispersal is fundamental to biogeography and the evolution of biodiversity on oceanic islands. *Journal of Biogeography* **33**, 193-198, doi:10.1111/j.1365-2699.2005.01383.x (2006).
84 Yang, S. F., Komaki, S., Brown, R. M. & Lin, S. M. Riding the Kuroshio current: stepping stone dispersal of the Okinawa tree lizard across the East Asian Island Arc. *Journal of Biogeography* **45**, 37-50 (2018).
85 Borreda, C., Perez-Roman, E., Ibanez, V., Terol, J. & Talon, M. Reprogramming of retrotransposon activity during speciation of the genus *Citrus*. *Genome Biol Evol* **11**, 3478-3495, doi:10.1093/gbe/evz246 (2019).
86 Talon, M., Wu, G. A., Gmitter, F. G. & Rokhsar, D. S. in *The Genus Citrus* (eds M. Talon, M. Caruso, & F.G. Gmitter) Ch. 2, 9-31 (Elsevier, 2020).
