# Supplementary material for: Diversification of mandarin citrus by hybrid speciation and apomixis
Source: Nat Commun. 2021 Jul 26;12:4377. doi: 10.1038/s41467-021-24653-0 (PMC8313541; doi:10.1038/s41467-021-24653-0)
Supplement: Supplementary file 6 — Description of additional supplementary files [file 41467_2021_24653_MOESM6_ESM.docx]

Description of additional supplementary information

Title: Supplementary Data 1.

Description: List of samples sequenced in this study.

Title: Supplementary Data 2.

Description: Additional information on citrus samples sequenced in this sudy.

Title: Supplementary Data 3.

Description: List of previously published resequencing data used in this study.
