# Supplementary material for: Diversification of mandarin citrus by hybrid speciation and apomixis
Source: Nat Commun. 2021 Jul 26;12:4377. doi: 10.1038/s41467-021-24653-0 (PMC8313541; doi:10.1038/s41467-021-24653-0)
Supplement: Supplementary file 7 — Reporting Summary [file 41467_2021_24653_MOESM7_ESM.pdf]

nature portfolio

Corresponding author(s): Guohong Albert Wu, Daniel S. Rokhsar, Frederick G. Gmitter Jr

Last updated by author(s): Jun 19, 2021

Reporting Summary

Nature Portfolio wishes to improve the reproducibility of the work that we publish. This form provides structure for consistency and transparency in reporting. For further information on Nature Portfolio policies, see our Editorial Policies and the Editorial Policy Checklist.

Statistics

For all statistical analyses, confirm that the following items are present in the figure legend, table legend, main text, or Methods section.

n/a	Confirmed	
☐	☒	The exact sample size (*n*) for each experimental group/condition, given as a discrete number and unit of measurement
☒	☐	A statement on whether measurements were taken from distinct samples or whether the same sample was measured repeatedly
☐	☒	The statistical test(s) used AND whether they are one- or two-sided<br>*Only common tests should be described solely by name; describe more complex techniques in the Methods section.*
☒	☐	A description of all covariates tested
☒	☐	A description of any assumptions or corrections, such as tests of normality and adjustment for multiple comparisons
☐	☒	A full description of the statistical parameters including central tendency (e.g. means) or other basic estimates (e.g. regression coefficient) AND variation (e.g. standard deviation) or associated estimates of uncertainty (e.g. confidence intervals)
☐	☒	For null hypothesis testing, the test statistic (e.g. *F*, *t*, *r*) with confidence intervals, effect sizes, degrees of freedom and *P* value noted<br>*Give P values as exact values whenever suitable.*
☒	☐	For Bayesian analysis, information on the choice of priors and Markov chain Monte Carlo settings
☒	☐	For hierarchical and complex designs, identification of the appropriate level for tests and full reporting of outcomes
☒	☐	Estimates of effect sizes (e.g. Cohen's *d*, Pearson's *r*), indicating how they were calculated

Our web collection on statistics for biologists contains articles on many of the points above.

Software and code

Policy information about availability of computer code

Data collection	no software was used
Data analysis	Read mapping and variant calling: BWA-MEM v0.7.8-r455, PICARD MarkDuplicates (version 2.13.2), GATK HaplotypeCaller (version 3.7-0-gcfedb67). For multidimensional scaling: R (version 3.5.1) function cmdscale . For phylogenomic inference: RAxML v8.2.12. Demographic inference: moments v1.0.3. Ancestry components size: ADMIXTURE v1.3.0. Fst estimate: vcftools v0.1.15. Custom wrapper scripts for demographic inference using moments are available at Github [https://github.com/citruscompgen/RyukyuCitrus.git]

For manuscripts utilizing custom algorithms or software that are central to the research but not yet described in published literature, software must be made available to editors and reviewers. We strongly encourage code deposition in a community repository (e.g. GitHub). See the Nature Portfolio guidelines for submitting code & software for further information.

Data

Policy information about availability of data

All manuscripts must include a data availability statement. This statement should provide the following information, where applicable:

- Accession codes, unique identifiers, or web links for publicly available datasets
- A description of any restrictions on data availability
- For clinical datasets or third party data, please ensure that the statement adheres to our policy

Data supporting the findings of this work are available within the paper and its Supplementary Information files. High coverage (average 46x) whole-genome shotgun-sequencing data of 69 citrus accessions generated in this study have been deposited at NCBI under BioProject PRJNA670310, with summary information for each accession in Supplementary Data 1 and 2. Previously published resequencing data used in this study are listed in Supplementary Data 3. Source data are provided with this paper.

Field-specific reporting

Please select the one below that is the best fit for your research. If you are not sure, read the appropriate sections before making your selection.

☐ Life sciences ☐ Behavioural & social sciences ☒ Ecological, evolutionary & environmental sciences

For a reference copy of the document with all sections, see nature.com/documents/nr-reporting-summary-flat.pdf

Ecological, evolutionary & environmental sciences study design

All studies must disclose on these points even when the disclosure is negative.

Study description	Genetic diversity and population structure of East Asian citrus
Research sample	Leafs from wild and cultivated citrus trees in Okinawa of the Ryukyu Islands and mainland Japan, including Citrus depressa, Citrus tachibana, and other indigenous citrus types grown in Okinawa. To investigate evolutionary relationships of East Asian citrus, published citrus resequencing data from mainland Asia were also used as listed in Supplementary Data 3.
Sampling strategy	We collected 69 new citrus samples for resequencing, with extensive sampling in Okinawa citrus growing areas. The issue of sample size is not relevant here, as the collection was generated to seek evolutionary relationships among the samples, not to estimate specific parameters of the population by sampling.
Data collection	Whole genome shot-gun DNA sequencing was performed by Okinawa Institute of Science and Technology Sequencing Center managed by Ms. Nana Arakaki.
Timing and spatial scale	Sample collection and DNA sequencing was performed during 2017-2019. Specific dates and frequency of collection is not relevant: these are long-lived trees whose leaves were collected, and sequenced DNA is stable and does not change over time.
Data exclusions	No data were excluded from the analyses.
Reproducibility	Our findings are computational, not "experimental".
Randomization	This is not relevant to our study, as our approach is computational. To study evolutionary relationships among the samples, we analyzed every sample in our collection in the context of published sequences.
Blinding	We did not consider the identities of the samples in our analyses. Instead, we infer sample identity by comparative genome analysis. In this sense, our study is blinded.

Did the study involve field work? ☒ Yes ☐ No

Field work, collection and transport

Field conditions	Environmental conditions were not recorded but are not relevant to our study, since we collected genomic DNA.
Location	Samples were collected at various places in Okinawa and mainland Japan as detailed in Supplementary Data 2.
Access & import/export	Permit to access one location in Okinawa was issued on April 25, 2019 by the Ministry of the Environment of Japan, with additional details in Supplementary Note 2.
Disturbance	None

Reporting for specific materials, systems and methods

We require information from authors about some types of materials, experimental systems and methods used in many studies. Here, indicate whether each material, system or method listed is relevant to your study. If you are not sure if a list item applies to your research, read the appropriate section before selecting a response.

Materials & experimental systems

n/a	Involved in the study
☒	☐ Antibodies
☒	☐ Eukaryotic cell lines
☒	☐ Palaeontology and archaeology
☒	☐ Animals and other organisms
☒	☐ Human research participants
☒	☐ Clinical data
☒	☐ Dual use research of concern

Methods

n/a	Involved in the study
☒	☐ ChIP-seq
☒	☐ Flow cytometry
☒	☐ MRI-based neuroimaging
